# Prevalence and risk factors of *Klebsiella* spp. in milk samples from dairy cows with mastitis—A global systematic review

**DOI:** 10.3389/fvets.2023.1143257

**Published:** 2023-03-22

**Authors:** Jinming Song, Wentao Xiang, Qi Wang, Jiying Yin, Tian Tian, Qizhu Yang, Meng Zhang, Guiyang Ge, Jianming Li, Naichao Diao, Fei Liu, Kun Shi, Ruopeng Cai, Rui Du, Qinglong Gong

**Affiliations:** ^1^College of Animal Science and Technology, Jilin Agricultural University, Changchun, China; ^2^College of Chinese Medicine Materials, Jilin Agricultural University, Changchun, China

**Keywords:** meta-analysis, bovine mastitis, *Klebsiella*, multidrug-resistance (MDR), prevalence

## Abstract

**Introduction:**

The overall prevalence of *Klebsiella* spp., a group of important zoonotic pathogens, in the global dairy herds and the risk of cross-species transmission between humans and dairy cows remain to be clarified. This systematic review aimed to determine the prevalence of *Klebsiella* spp. in milk samples from dairy cows with mastitis worldwide and to assess the factors influencing the prevalence of these strains.

**Methods:**

Qualified studies published from 2007 to 2021 were retrieved from ScienceDirect, Web of Science, PubMed, WanFang Database, China National Knowledge Infrastructure (CNKI), and VIP Chinese Journal Database. Calculations of prevalence and their 95% confidence intervals (CIs) were performed for all the studies using the Freeman-Tukey double arcsine transformation (PFT).

**Results:**

A total of 79,852 milk samples from 55 manuscripts were examined in this meta-analysis, and 2,478 samples were found to be positive for *Klebsiella* spp. The pooled prevalence estimates worldwide were 7.95% (95% CI: 6.07%–10.06%), with significant heterogeneity (*I*^2^ = 98.8%, *p* = 0). The sampling period of 2013–2020 had a higher (*p* < 0.05) Klebsiella-positive proportion of milk samples (12.16%, 95% CI: 8.08%–16.90%) than that of 2007–2012 (3.85%, 95% CI: 2.67%–5.21%), indicating that bovine mastitis caused by Klebsiella may become increasingly prevalent. The risk factors for the high prevalence of Klebsiella in milk samples mainly included: economic development level (developing countries; 11.76%, 95% CI: 8.25%–15.77%), mastitis type (CM; 11.99%, 95% CI: 8.62%–15.79%), and population density (>500 per sq km; 10.28%, 95% CI: 2.73%–21.58%). Additionally, a bivariate meta-regression analysis revealed that the multidrug-resistance (MDR) rate of the epidemic strains was also closely related to economic development level (*R*^2^ = 78.87%) and population density (*R*^2^ = 87.51%).

**Discussion:**

Due to the potential risk of cross-species transmission between humans and cows, the prevalence of mastitis milk-derived Klebsiella and its high MDR rate need to be monitored, especially in developing countries with high population densities.

## Introduction

As one of the common diseases in dairy farming, bovine mastitis can lead to decreased milk production, poor milk quality, reproductive barriers and even culling of dairy cows, causing great economic losses to the global dairy industry (1). Bovine mastitis is classified as clinical mastitis (CM) and subclinical mastitis (SCM) according to symptoms and milk characteristics. In general, CM is defined as a condition showing swelling of the infected quarter and in some cases systemic signs such as fever and anorexia. However, it is difficult to detect SCM visually unless through diagnostic techniques such as the California Mastitis Test (CMT), Wisconsin Mastitis Test (WMT) or Somatic Cell Count (SCC) (2). Due to the complexity of the bacteria that cause intramammary infection (IMI) in dairy cows, the control of bacterial infection is mainly performed with antibiotic management and assisted by the treatment of some bacteriophages and natural compounds at present. Moreover, some vaccines have been developed to prevent bovine mastitis (1).

As a common zoonotic pathogen that is widely prevalent in hospitals and communities, *Klebsiella pneumoniae* mainly causes pneumonia, liver abscess, urinary diseases, toxemia, septicaemia and other infection symptoms (3). Due to the long-term irrational use of antibiotics in the medical field, a wide variety of carbapenemase [such as *K. pneumoniae* carbapenemase (KPC), New Delhi metallo-β-lactamase (NDM) and oxacillinase (OXA)]-producing and extended spectrum β-lactamase (ESBL, such as CTX-M, SHV and TEM)-producing multidrug-resistant (MDR) *Klebsiella* have emerged in an endless number of cases (4). *Klebsiella* spp., as vehicles of multiple drug-resistance genes, are monitored by the World Health Organization (WHO) (5). Under the pressure of antibiotic selection, drug-resistance genes are transferred to other strains through mobile genetic elements (MGEs) such as plasmids, transposons, and insertion elements, thus increasing the challenge of mitigating bacterial infections (6). *Klebsiella* spp. is also an important cause of bovine mastitis, with *K. pneumoniae* and *K. oxytoca* as the most prevalent species (7, 8). In dairy cattle, *Klebsiella* spp. are transmitted by contact with teats, mainly through manure, bedding and other farm equipment, and invade mammary epithelial cells and persist for a long time (9). Moreover, these strains not only seriously affect the milk quality and performance of adult cows, but also pose a fatal threat to the survival of newborn calves (10). To the best of our knowledge, bovine mastitis caused by *K. pneumoniae* was first publicly reported in 1954 (11), but in the following decades, the prevalence of *Klebsiella* in milk samples appeared to be nonsignificant compared to that of *Staphylococcus aureus, Streptococcus agalactiae*, and *Escherichia coli*. However, over the past decade or so, the number of cases of *Klebsiella* spp. detected in milk samples has risen dramatically worldwide. In some countries, these bacteria are second only to *E. coli* in the incidence of gram-negative bacteria in dairy cow udders (8).

The global spread and distribution of carbapenemase-producing and hypervirulent *Klebsiella* spp. from human sources in the past decade have been revealed (12, 13). During the same period, reports of *Klebsiella* spp. being detected in raw milk samples from dairy farming regions (especially Holstein-Friesian dairy farms) around the world have increased. Although *K. pneumoniae* isolated from companion animals shows zoonotic potential (14, 15), there is currently no assessment of risk factors for cross-species transmission of these strains in humans and dairy herds due to the lack of systematic analysis of the global distribution of milk-derived *Klebsiella* spp. To fully evaluate the global prevalence of *Klebsiella* spp. in milk samples from dairy cows with mastitis and to assess risk factors for influencing the prevalence of these strains, this systematic review and meta-analysis was conducted on the prevalence of *Klebsiella* spp. in milk samples from major Holstein dairy farming regions using articles published from 2007 to 2021 based on subgroups (sampling years, detection methods, geographic information, level of economic development, mastitis type, population density, *Klebsiella* species, and MDR rate of *Klebsiella* isolates).

## Materials and methods

### Study design and search strategies

We conducted a systematic literature search for studies that examined the prevalence of *Klebsiella* spp. in milk samples from dairy cows worldwide following the Preferred Reporting Items for Systematic Reviews and Meta-Analyses (PRISMA) guidelines (16). On January 12, 2022, six databases were searched: ScienceDirect, Web of Science (all databases), PubMed, WanFang Database, China National Knowledge Infrastructure (CNKI), and VIP Chinese Journal Database. We found that the search items “Klebsiella” and “Mastitis” produced the most qualified studies by presearch.

The keywords “Klebsiella” and “Mastitis” were used in the process of screening ScienceDirect, and “Research articles” was chosen as the article type. Web of Science was searched by combining two queries #1 “TS = (Mastitis)” and #2 “TS = (Klebsiella)” with “AND”. In PubMed, we used the MeSH terms “Klebsiella” and “Mastitis” in an advanced search to generate the search formula (Klebsiella) AND (Mastitis). In the WanFang database, CNKI, and the VIP Chinese Journal database, the topics were defined as “Mastitis” AND “Klebsiella” in Chinese in the advanced search. Considering that some qualified studies may have not been included in the electronic database we built, all the references cited in relevant studies were carefully checked. Subsequently, the supplementary search was conducted using Google Scholar, which allowed those missing articles with available data to be included in this meta-analysis. In addition to English and Chinese studies, a Spanish study and a Korean study (both with English abstracts) were included through a supplementary search. Records identified in the search process were uploaded into EndNote (version X 9.3.3).

### Selection criteria

We preliminarily screened articles based on duplication, and those with duplicate titles and abstracts were excluded with the help of Endnote. After title/abstract screening, full texts were screened afterwards. Qualified studies were selected according to the following criterion: articles which were published between 2007 and 2021 that examined the prevalence of *Klebsiella* spp. in milk samples. Studies were excluded if they met the following criteria: (1) the full text was unavailable, (2) sample totals or prevalence were not provided, (3) the sampling time was unclear, and (4) the samples collected were not milk.

Two authors (JS and WX) performed the selection process according to the eligibility criteria.

### Data extraction

Standardized forms generated by Microsoft Excel 2019 (version 2203) were used to extract the following information from the eligible studies: first author, publication year, sampling years, sample size, number of *Klebsiella*-positive milk samples, detection methods, geographic information, mastitis type, economic development level and population density of the country where the study was conducted, and the MDR rate of *Klebsiella* spp. isolated from milk samples. The milk samples included were all individual quarter samples.

In 2013, MDR *K. pneumoniae* was listed by the Centers for Disease Control and Prevention (CDC) as an urgent threat to public health, along with other carbapenem-resistant *Enterobacteriaceae* (CRE) (17). Therefore, we chose 2013 as a time point to divide the timeline into two parts to conduct a systematic review and meta-analysis.

The economic development levels of the countries were established according to World Economic Situation and Prospects 2022—UN (18). A list of countries by population density was derived from World Population Prospects 2022—UN (19).

### Quality assessment

The quality of each eligible article was estimated by scoring (20, 21). In brief, the following items were given 1 point when they were present in a study: random sampling; explicit detection method; detailed sampling procedures; sampling year; and risk factors ≥4. The articles were scored with a range of 0 to 5, and they were divided into three intervals: 0–1 points, 2–3 points, and 4–5 points. The scoring criteria were only applicable to this meta-analysis and did not represent the research level of the included studies.

### Statistical analysis

All the data were analyzed by the package “meta” (version 4.0.0) in R software version 4.0.0 (R Foundation for Statistical Computing, Vienna, Austria). The prevalence of *Klebsiella*-positive milk samples was estimated as the number of milk samples in which *Klebsiella* spp. was detected divided by the number of total tested milk samples. Calculations of prevalence and their 95% confidence intervals (CIs) were performed for all the studies. The Freeman-Tukey double arcsine transformation (PFT) showed better variance stabilization performance by normalizing the data with different meta packages previously. Therefore, to make the distribution in accordance with Gaussian distribution, we performed rate conversion with PFT. The formulates for PFT were as follows (22):


$$\begin{array}{l} \;t=arc\sin\left(sqrt\left(\frac{r}{n+1}\right)\right)+arc\sin\left(sqrt\left(\frac{r+1}{n+1}\right)\right)\\ se\left(t\right)=sqrt\;\left(\frac{1}{n+0.5}\right)\\ \;p={\left(\sin\frac{t}{2}\right)}^{2} \end{array}$$

*Note*: *t* = *transformed prevalence*; *n* = *sample size*; *r* = *positive number*; *se* = *standard error*.

The transformed summary proportion and its confidence interval were reconverted for better readability. The *I*^2^, Cochran's Q, and χ^2^ tests were used to quantify the variation (23, 24). Because of the high degree of heterogeneity, we conducted the meta-analysis using a random effects model. Publication bias (indicated by symmetry of the funnel plot) was evaluated by performing a funnel plot, trim and fill method, and Egger's test. A *p* < 0.05 was considered to be the statistical significance threshold (25, 26). Funnel plots were generated to further assess every subgroup. To determine the stability of the study, we also conducted sensitivity analysis, which evaluated the effect of each study on the overall results by sequentially excluding single studies. The R code for this meta-analysis is shown in Supplementary Table 1.

Subgroup analysis and univariate meta-regression analysis were used to reveal factors that may contribute to heterogeneity among studies. The independent factors that we selected were sampling years (comparison of 2013 or later with before 2013), detection methods (comparison of automatic analysis systems with other methods), continents (comparison of Oceania with other continents), longitude (comparison of 0–20°E with other intervals), latitude (comparison of 20–30°N with other intervals), economic development level (comparison of developing countries with developed countries), mastitis type (comparison of CM with SCM), population density (comparison of “ < 50 per sq km” with other groups), and *Klebsiella* species (comparison of *K. pneumoniae* with *K. oxytoca*). In addition, we performed a bivariate meta-regression analysis to analyze the correlation of the MDR rate of *Klebsiella* spp. isolated from milk samples with population density and economic development level, and R^2^ was used to explain the heterogeneity of each term by indicating the proportion (27).

## Results

### Search results and eligible studies

**Figure 1:**
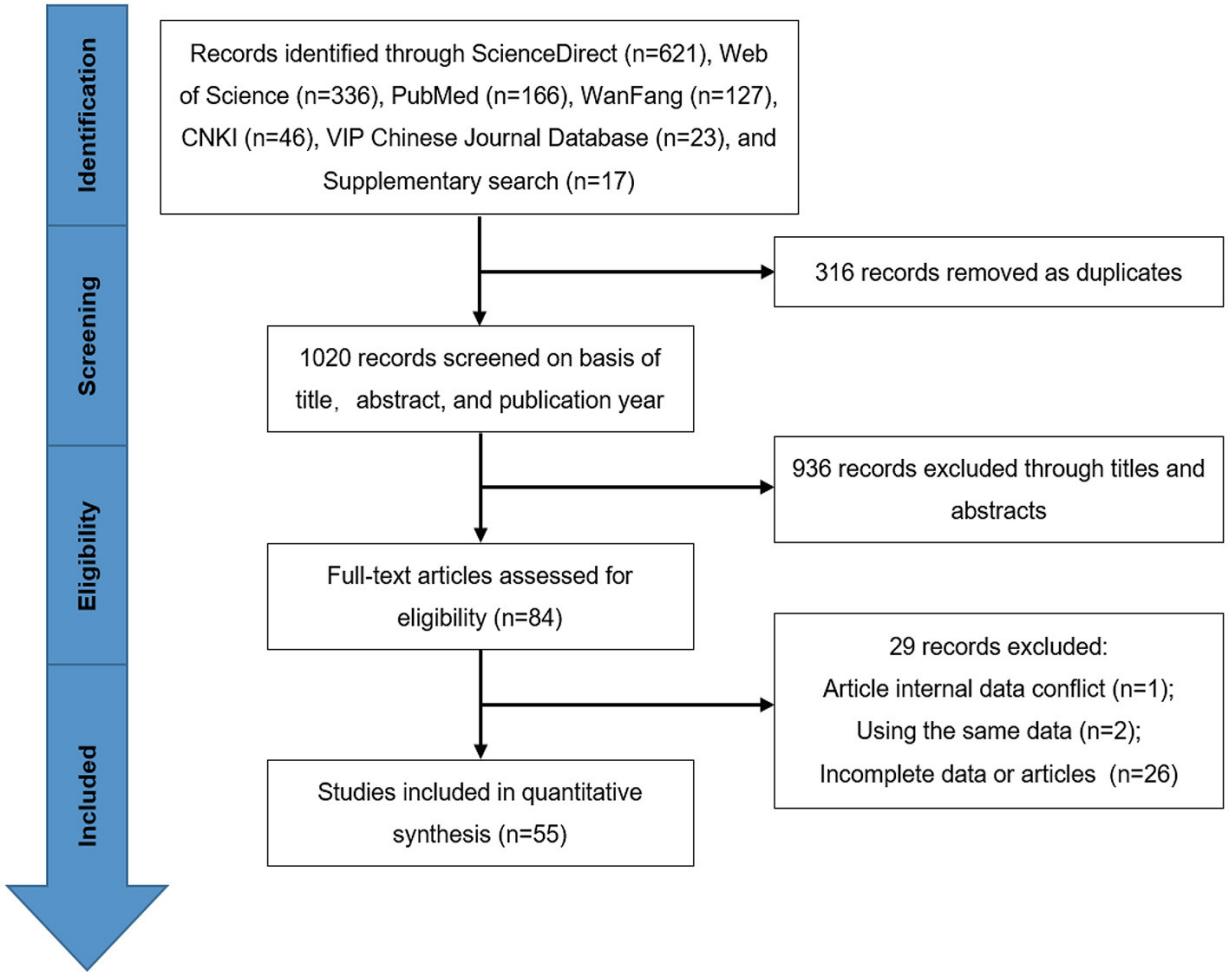
Flow diagram of search and selection process of eligible studies.

**Figure 2:**
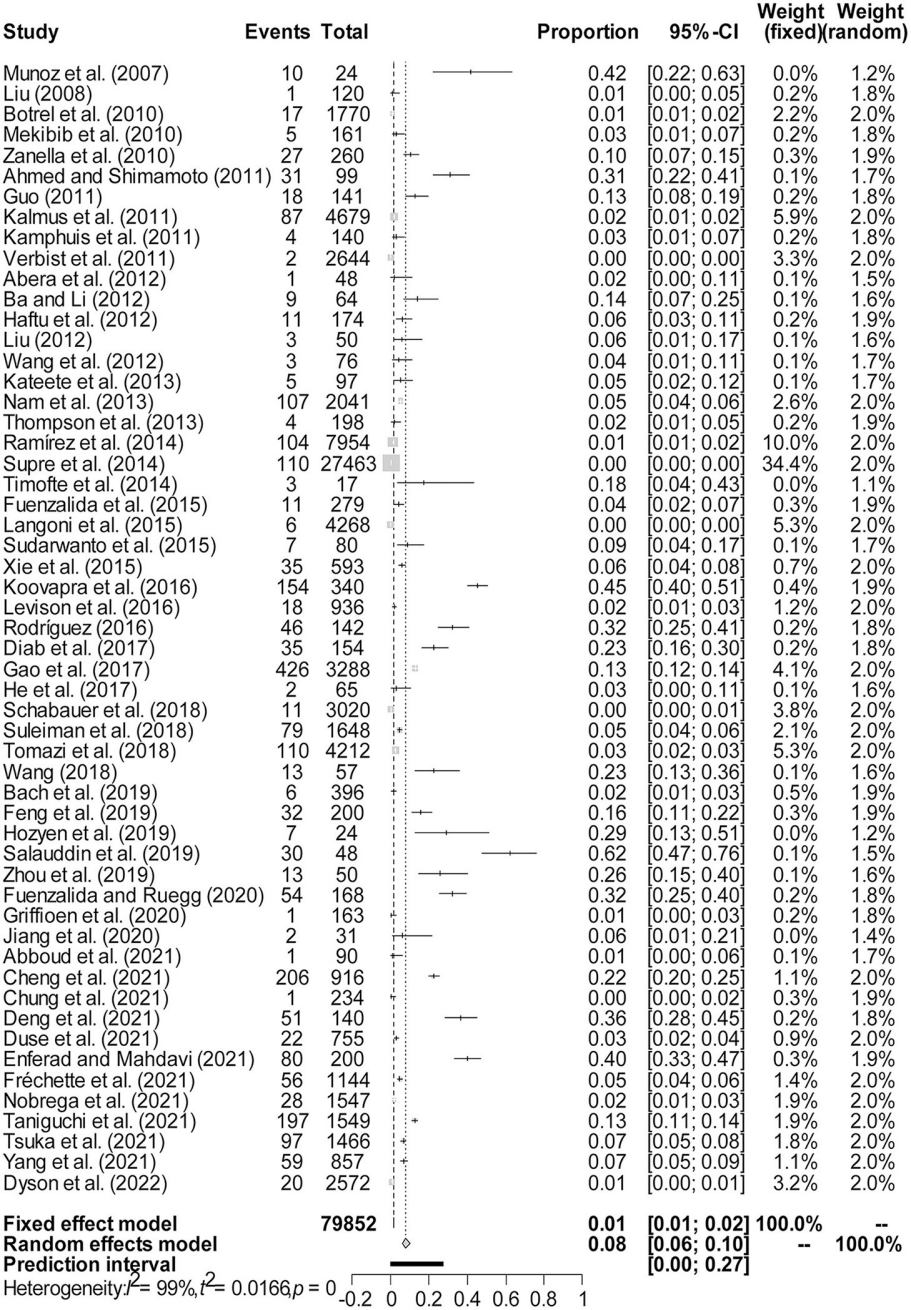
Forest plot of the prevalence of *Klebsiella* spp. in milk samples among studies conducted worldwide. The length of the horizontal line represents the 95% confidence interval, and the diamond represents the summarized effect.

A total of 1,319 records were identified by searching the databases. First, 316 duplicate studies were removed. According to the previously established inclusion criterion, 936 studies were excluded because failed to meet publication years (2007–2021) or targeted objectives (*Klebsiella* spp. in milk samples from dairy cows with mastitis) through screening of the titles and abstracts. After that, the full texts of the remaining 84 studies were derived for further assessment based on the inclusion criterion. Finally, we included 55 studies in this meta-analysis (Figures 1, 2). In terms of quality, 14 studies scored 2 or 3 points, and 41 studies scored 4 or 5 points (Supplementary Table 2).

### Publication bias and sensitivity analysis

**Figure 3:**
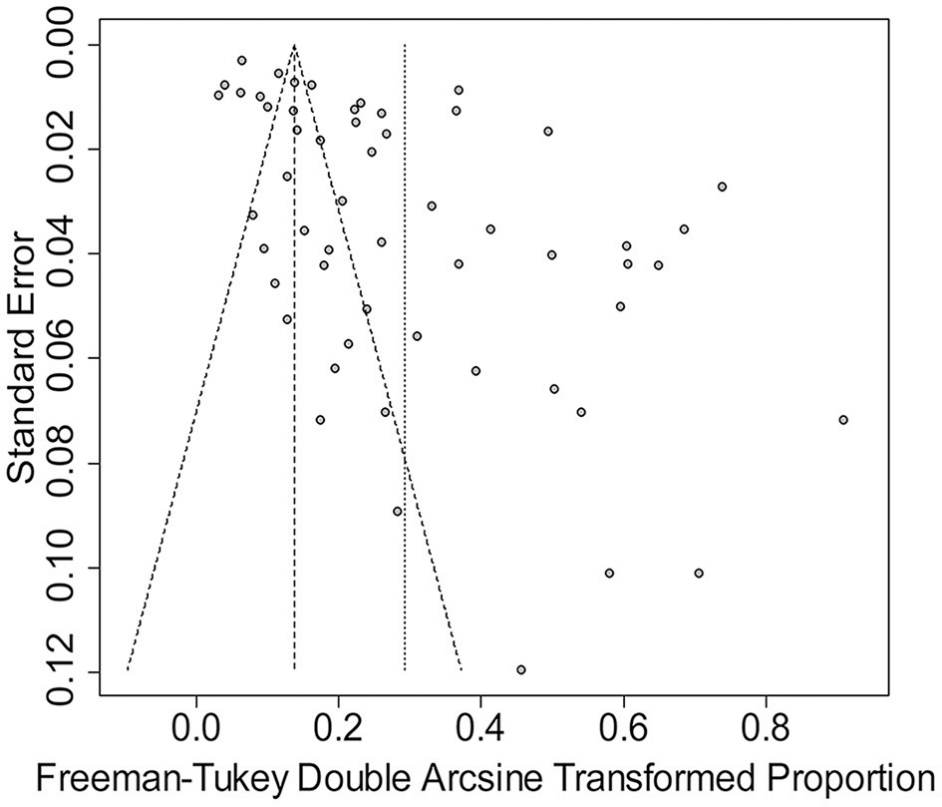
Funnel plot with pseudo 95% confidence interval limits for the examination of publication bias.

**Figure 4:**
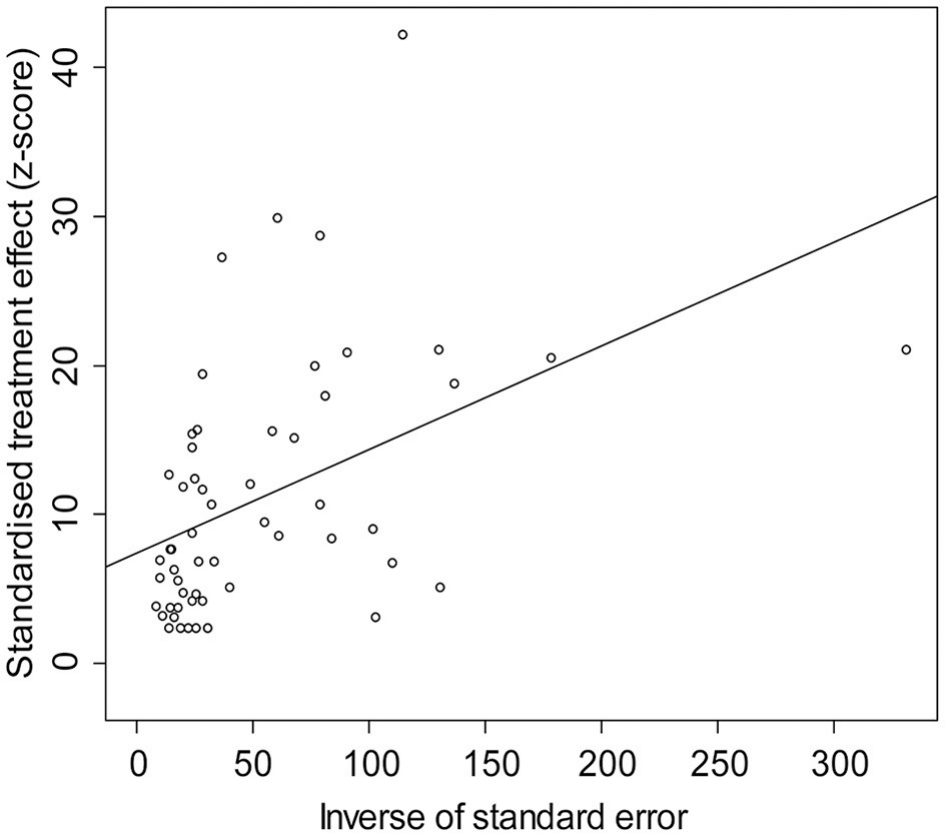
Egger's test for publication bias.

**Figure 5:**
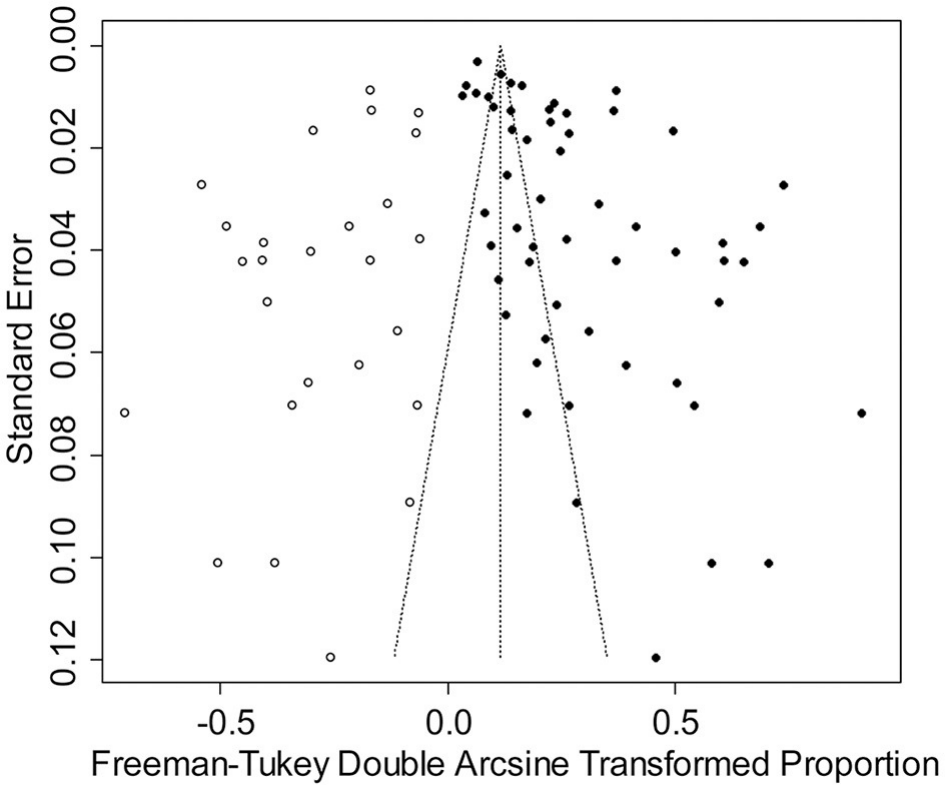
The trim and fill method for publication bias.

**Figure 6:**
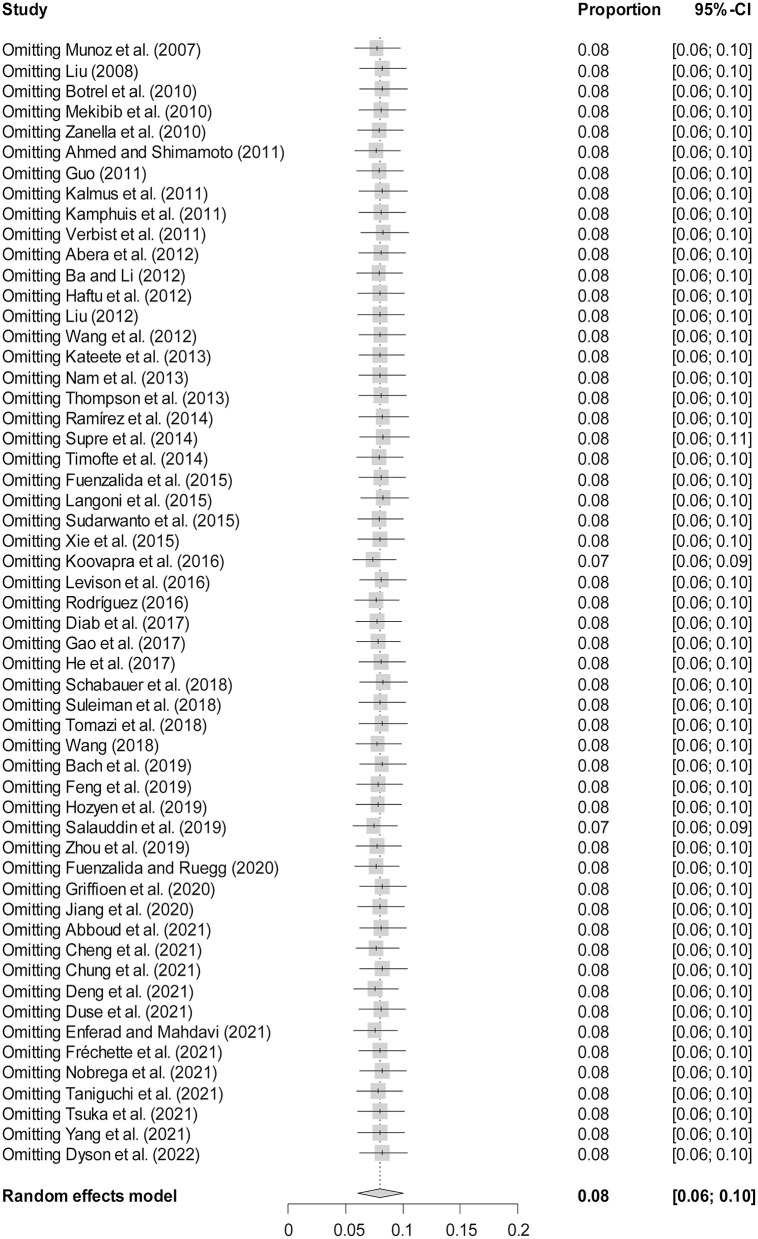
Sensitivity analysis. After removing one study at a time, the remaining studies were recombined using a random effects model to verify the effect of a single study on the overall results.

The rate was converted using PFT to ensure that the distribution was more similar to a normal distribution (Table 1). The forest plot revealed a high heterogeneity among the included studies (*I*^2^ = 98.8%, *p* = 0; Figure 2). The funnel plot was not symmetrical, indicating publication bias or small sample size bias in the studies (Figure 3). Egger's test was used to further test the sources of funnel plot asymmetry and provided a *p*-value of < 0.001 (Figure 4, Supplementary Table 3), indicating that publication bias did exist. The publication bias disappeared after adding 26 studies (the point estimate was 0%) when evaluating publication bias by the trim and fill method (Figure 5). Furthermore, we performed a sensitivity analysis to determine the effect of each study on the pooled prevalence of *Klebsiella* spp. There was no significant change in the result after excluding individual studies, which indicated the stability of our meta-analysis (Figure 6).

**Table 1:** Normal distribution test for the normal rate and the different conversions of the normal rate.

**Conversion form**	***W***	***P***
PRAW^a^	0.77945	1.12e-07
PLN^b^	0.96893	0.1648
PLOGIT^c^	0.98594	0.7657
PAS^d^	0.90654	0.0004236
PFT^e^	0.90207	0.0002939

^a^PRAW: original rate.

^b^PLN: logarithmic conversion.

^c^PLOGIT: logit transformation.

^d^PAS: arcsine transformation.

^e^PFT: double-arcsine transformation.

The meta-analysis results and publication bias of each subgroup are shown in Supplementary Figures 1–8.

### Pooled prevalence of *Klebsiella* spp. in milk samples

There were 79,852 milk samples tested in our meta-analysis, and 2,478 of them were found to contain *Klebsiella* spp. The majority of *Klebsiella* species isolated from the milk of cows with mastitis is *K. pneumoniae* (25/55), followed by *K. oxytoca* (6/55), and only one case of *K. ozaenae* has been reported (Supplementary Table 4). At the global level, the random effect estimated pooled prevalence was 7.95% (95% CI: 6.07%−10.06%), and there was substantial heterogeneity (*x*^2^ = 4584.55, *I*^2^ = 98.8%, *p* = 0). The prevalence of *Klebsiella*-positive milk samples is shown in Figure 2.

The pooled *Klebsiella* spp. prevalence estimates and the 95% CIs for corresponding subgroups are all reported in Table 2. The pooled prevalence of *Klebsiella-*positive samples detected in 2013 or later was 12.16% (95% CI: 8.08%−16.90%), which was higher (*p* < 0.05) than that obtained before 2013 (3.85%, 95% CI: 2.67%−5.21%). By comparing various detection methods, automatic analysis systems (including the API 20E System and VITEK 2 Automated Identification System) showed the highest prevalence (17.14%, 95% CI: 10.33%−25.17%). The pooled prevalence estimates of *K. oxytoca* (3.22%, 95% CI: 0.73%−7.15%) in milk samples were lower than that of *K. pneumoniae* (11.51%, 95% CI: 6.72%−17.31%). However, considering *p* > 0.05 and small number of studies and samples of *K. oxytoca*, there existed a small sample size bias which indicated the results of this subgroup were not robust.

**Table 2:** Pooled prevalence of *Klebsiella* spp. in milk samples from dairy cows with mastitis worldwide including risk factors and meta-regression.

		**No. studies**	**No. tested**	**No. positive**	**Positive percentage (95% CI^a^)**	**Heterogeneity**			**Univariate meta-regression**	
						χ^2^	***p*** **-value** ^b^	***I***^2^ **(%)**	***p*** **-value**	**Coefficient (95% CI)**
Sampling years									0.0004	0.1339 (0.0599–0.2079)
	Before 2013	25	26,171	536	3.85% (2.67–5.21%)	505.76	< 0.01	94.9%		
	2013 or later	28	24,752	1,735	12.16% (8.08–16.90%)	2,725.28	0	99.0%		
Detection methods									0.0099	0.1565 (0.0376–0.2754)
	Biochemical tests	32	64,699	1,626	6.97% (4.93–9.31%)	2,705.81	0	98.9%		
	16SrDNA identification	9	12,770	806	11.04% (4.01–20.82%)	1,550.65	0	99.5%		
	MALDI-TOF MS	8	4,154	315	4.56% (1.51–9.01%)	188.45	< 0.01	96.3%		
	Automatic analysis systems	6	5,090	615	17.14% (10.33–25.17%)	126.76	< 0.01	96.1%		
Continents									0.00240	−0.2169 (−0.4053 to −0.0285)
	Africa	7	2,251	139	8.72% (3.75–15.32%)	67.02	< 0.01	91.0%		
	Asia	24	12,616	1,581	14.26% (10.49–18.48%)	789.51	< 0.01	97.1%		
	Europe	9	40,651	257	0.73% (0.20–1.49%)	159.78	< 0.01	95.0%		
	North America	7	3,145	159	7.52% (2.99–13.73%)	160.46	< 0.01	96.3%		
	South America	6	18,383	321	4.48% (2.14–7.61%)	325.69	< 0.01	98.5%		
	Oceania	2	2,806	21	0.67% (0.38–1.03%)	0.10	0.76	0.0%		
Longitude									< 0.0001	−0.2229 (−0.3097 to −0.1362)
	0**–**20°E	7	35,955	167	0.70% (0.28–1.26%)	64.72	< 0.01	90.7%		
	20**–**40°E	10	7,174	262	7.72% (4.00–12.42%)	199.63	< 0.01	95.5%		
	40**–**60°E	1	200	80	40.00% (33.30–46.89%)	0.00	—	—		
	80**–**100°E	1	48	30	62.50% (48.27–75.75%)	0.00	—	—		
	100**–**120°E	18	7,580	649	10.11% (7.43–13.13%)	209.11	< 0.01	91.9%		
	140**–**160°E	1	2,572	20	0.78% (0.47–1.16%)	0.00	—	—		
	0**–**20°W	2	447	65	15.23% (0.00–50.98%)	67.24	< 0.01	98.5%		
	40**–**60°W	3	6,075	61	2.60% (0.15–7.67%)	113.26	< 0.01	98.2%		
	60**–**80°W	4	8,516	166	12.59% (2.29–28.79%)	168.56	< 0.01	98.2%		
	80**–**100°W	2	953	21	6.22% (0.00–29.58%)	6.81	< 0.01	85.3%		
Latitude									0.0001	0.2421 (0.1189–0.3652)
	0**–**10°S	3	1,870	132	13.37% (1.30–34.48%)	78.94	< 0.01	97.5%		
	10**–**20°S	1	1,547	28	1.81% (1.20–2.54%)	0.00	—	—		
	20**–**30°S	2	4,528	33	3.23% (0.00–20.37%)	83.49	< 0.01	98.8%		
	30**–**40°S	1	2,454	19	0.77% (0.46–1.16%)	0.00	—	—		
	40**–**50°S	1	118	1	0.85% (0.00–3.60%)	0.00	—	—		
	0**–**10°N	4	8,260	115	2.33% (0.63–4.86%)	9.52	0.02	68.5%		
	10**–**20°N	1	174	11	6.32% (3.12–10.48%)	0.00	—	—		
	20**–**30°N	4	415	84	24.13% (6.02–48.96%)	73.14	< 0.01	95.9%		
	30**–**40°N	16	7,375	719	12.91% (8.93–17.46%)	349.68	< 0.01	95.7%		
	40**–**50°N	10	6,918	155	6.20% (2.81–10.68%)	299.14	< 0.01	97.0%		
	50**–**60°N	6	35,106	207	0.60% (0.01–1.73%)	122.70	< 0.01	95.9%		
Economic development level									< 0.0001	0.1561 (0.0840–0.2282)
	Developed countries	21	51,658	838	3.31% (1.89–5.08%)	1,296.66	< 0.01	98.5%		
	Developing countries	34	28,194	1,640	11.76% (8.25–15.77%)	2,596.12	0	98.7%		
Mastitis type									0.0447	0.0987 (0.0023–0.1951)
	Clinical	31	16,990	1,233	11.99% (8.62–15.79%)	1,222.44	< 0.01	97.5%		
	Subclinical	13	14,627	420	6.42% (2.97–10.98%)	770.04	< 0.01	98.4%		
Population density									0.0141	−0.1021 (−0.1837 to −0.0206)
	< 50	17	29,768	610	4.13% (2.70–5.83%)	595.21	< 0.01	97.3%		
	50–100	2	1,848	159	19.00% (0.00–62.04%)	153.85	< 0.01	99.4%		
	100**–**250	24	12,121	968	9.58% (5.64–14.37%)	1,222.15	< 0.01	98.1%		
	250–500	6	33,479	563	8.88% (2.18–19.07%)	1,336.36	< 0.01	99.6%		
	>500	6	2,636	178	10.28% (2.73–21.58%)	149.23	< 0.01	96.6%		
Scores of study quality									0.8368	−0.0087 (−0.0920 to 0.0745)
	2–3	14	39,846	572	8.20% (4.72–12.49%)	1,222.17	< 0.01	98.9%		
	4–5	41	40,006	1,906	7.88% (5.67–10.39%)	2,643.54	0	98.5%		
Total		55	79,852	2,478	7.95% (6.07–10.06%)	4,584.55	0	98.8%		

^a^CI, confidence interval.

^b^*p*-value: *p* < 0.05 is statistically significant.

The 55 eligible studies were from 25 countries on six continents (Asia: 24; Europe: 9; Africa: 7; North America: 7; South America: 6; Oceania: 2). The prevalence of *Klebsiella* spp. in Asia was the highest (14.26%, 95% CI: 10.49%−18.48%), particularly in the longitude interval 40–100°E. The 0–20°E interval, where most European countries included in this meta-analysis were located, had the lowest positive proportion. In regard to latitude, there was a low *Klebsiella* prevalence at high latitudes. Almost all countries in the European (0.73%, 95% CI: 0.20–1.49%) and Oceanian (0.67%, 95% CI: 0.38–1.03%) regions are developed countries, which indicates that the distribution of the *Klebsiella*-positive proportion may be related to the level of national economic development.

The pooled prevalence of *Klebsiella* spp. samples detected in developing countries was 11.76% (95% CI: 8.25–15.77%), which was higher (*p* < 0.05) than that in developed countries (3.31%, 95% CI: 1.89–5.08%). There were more (*p* < 0.05) *Klebsiella*-positive samples found in clinical mastitis samples (11.99%, 95% CI: 8.62–15.79%) than in subclinical mastitis samples (6.42%, 95% CI: 2.97–10.98%).

In addition, the prevalence of *Klebsiella* spp. was related to population density. The analysis of population density in different countries showed that the lowest *Klebsiella* spp. prevalence was in countries where there were < 50 per sq km (4.13%, 95% CI: 2.70–5.83%) and revealed that the higher the population density of the sampling area, the higher the prevalence of *Klebsiella* spp. in milk samples.

To further explore the MDR rate of *Klebsiella* spp. isolated from milk samples, an MDR rate correlation analysis was conducted for economic development level and population density. The R^2^ values were 78.87 and 87.51% (Table 3), respectively, which revealed that the MDR rate was closely related to the level of economic development and population density.

**Table 3:** The MDR rate of *Klebsiella* spp. isolated from milk samples: a bivariate meta-regression analysis.

	**No. studies**	**No. isolates^a^**	**No. MDR^b^**	**Positive percentage (95% CI)**	**Heterogeneity**			**Univariate meta-regression**		**Bivariate meta-regression**	
					χ^2^	***p*** **-value**	***I*** ^2^	***p*** **-value** ^c^	**Coefficient (95% CI)**	**R** ^2^ **–economic development level**	**R** ^2^ **–population density**
MDR rate								0.0214	−0.5828 (-1.0793 to−0.0863)	78.87%	87.51%
0	4	33	0	0.00% (0.00–0.00%)	0.89	0.83	0.0%				
>0, < 0.5	8	860	129	15.02% (5.85–26.97%)	103.38	< 0.01	93.2%				
> 0.5, < 1	2	112	73	63.13% (46.19–78.61%)	2.76	0.10	63.8%				
1	6	81	81	100.0% (100.0–100.0%)	1.38	0.93	0.0%				

^a^No. isolates: the number of *Klebsiella* isolates.

^b^No. MDR: the number of MDR *Klebsiella* isolates.

^c^*p*-value: *p* < 0.05 is statistically significant.

## Discussion

This study investigated the prevalence of *Klebsiella* spp. in milk samples from dairy cows with mastitis worldwide by analyzing articles published from 2007 to 2021 to assess risk factors for influencing the prevalence of these strains and to improve solutions for bovine intramammary infections. The studies included in this analysis from 2007 to 2013 mostly came from European and North American developed countries. However, the prevalence of *Klebsiella*-positive milk samples from the European Union (EU) has decreased dramatically after 2013 (Table 4). For example, the prevalence of *Klebsiella* in CM milk samples from the Netherlands during 2017–2018 was lower than that in 2006–2009 (28, 29), which might be due to strict health management regulations by the EU member countries (30). Because *Klebsiella* spp. in bovine mastitis mostly originates from the manure attached to the surrounding bedding and farming equipment, especially the organic bedding materials such as sawdust (31, 32). In developed countries, frequent changes in bedding, along with cow cleanliness and the hygiene of housing facilities greatly reduce the contact transmission of these bacteria (33). Although the *Klebsiella*-positive rate of CM milk samples from the United States and Japan was comparatively prominent after 2013, cases were mainly reported in developing countries and showed a global distribution, especially in Asia. For instance, the prevalence of *K. pneumoniae* in mastitis milk samples in West Bengal, Jharkhand and Mizoram in India was 45.29% (95% CI: 40.03–50.61%) (34). Random sampling of free-range dairy herds in northwestern Iran revealed that the *K. pneumoniae*-positive proportion was 40.00% (95% CI: 33.30–46.89%) (35). The prevalence in CM milk samples in Rangpur Division, Bangladesh was up to 62.50% (95% CI: 48.27–75.75%) (36). One distinguishing feature of these regions is that they have extremely high population densities, and Bangladesh and some regions in India each have a population density of over 1,000 per sq km (19). In addition, in these regions, milk is usually produced in rural settings where the majority of the population resides (37). We also noted a surge in cases from China in the past decade, which may be partly related to the selection of three Chinese databases for this study and the increasing attention to bovine mastitis in this country. As fecal shedding of *K. pneumoniae* plays a critical role in pathogen dissemination (38), fecal-oral transmission cycles may perpetuate and amplify the presence of such pathogens (32). Human feces are recognized as a reservoir of reverse transmission (39). Although the evidence for its role in the transmission of *Klebsiella* from humans to dairy herds is unclear, some latrines in close proximity to dairy herds should be considered a risk factor that can promote human-derived *Klebsiella* transmission, and this risk may be increased by excessive population density.

**Table 4:** Characteristics of the included studies.

**References**	**Sampling time**	**No. positive/No. tested^a^**	**Prevalence**	**Mastitis type**	**Country**	**Population density^b^**	**Detection methods^c^**	***Klebsiella* species^d^**	**Genotyping methods**	**Gene detection^e^**	**MDR rate^f^**
Munoz et al. (50)	2006.04	10/24	41.67%	Clinical	The United States	36.39	—	Kpn	RAPD	—	—
Liu (51)	2008	1/120	0.83%	Clinical	China	153.8	Biochem	Unknown Species	—	—	0/1
Botrel et al. (52)	2007.01–2008.03	17/1,770	0.96%	Both^g^	France	119.5	Biochem	Unknown Species	—	—	—
Mekibib et al. (53)	2008.11–2009.04	5/161	3.11%	Subclinical	Ethiopia	117.9	Biochem	Kpn	—	—	—
Zanella et al. (54)	2006.01–2007.06	27/260	10.38%	—	Brazil	25.6	Biochem	Kpn, Kox, and Koz	—	—	—
Ahmed and Shimamoto (48)	2008	31/99	31.31%	—	Egypt	104.7	Biochem and Auto	Kpn and Kox	—	Drug resistance	14/31
Guo (55)	2010	18/141	12.77%	Both	China	153.8	Biochem	Unknown Species	—	—	—
Kalmus et al. (56)	2007–2009	87/4,679	1.86%	Both	Estonia	31.26	Biochem	Unknown Species	—	—	—
Kamphuis et al. (28)	2011	4/140	2.86%	Clinical	The Netherlands	509.3	Biochem	Unknown Species	—	—	—
Verbist et al. (31)	2008.05–2009.05	2/2,644	0.08%	Clinical	Belgium	384.2	Biochem	Unknown Species	—	—	—
Abera et al. (57)	2008.10–2009.05	1/48	2.08%	—	Ethiopia	117.9	—	Unknown Species	—	—	—
Ba and Li (58)	2011.07–2011.08	9/64	14.06%	—	China	153.8	Biochem	Unknown Species	—	—	0/9
Haftu et al. (59)	2009.10–2010.05	11/174	6.32%	Both	Ethiopia	117.9	Biochem	Kpn	—	—	—
Liu (60)	2009.01–2009.12	3/50	6.00%	Subclinical	China	153.8	Biochem	Unknown Species	—	—	—
Wang et al. (61)	2011.03–2011.04	3/76	3.95%	Subclinical	China	153.8	Biochem	Unknown Species	—	—	—
Kateete et al. (62)	2010.02–2011.03	5/97	5.15%	Clinical	Uganda	235.8	Biochem	Kox	—	—	2/5
Nam et al. (63)	2012.01–2012.11	107/2,041	5.24%	—	Korea	527.7	Biochem	Unknown Species	—	—	—
Thompson et al. (64)	2007.06–2008.08	4/198	2.02%	Clinical	Canada	4.168	—	Unknown Species	—	—	—
Ramírez et al. (65)	2009.01–2010.12	104/7,954	1.31%	Subclinical	Colombia	46.21	Biochem	Unknown Species	—	—	—
Supre et al. (66)	2012.09–2013.09	110/27,463	0.40%	—	Belgium	384.2	Biochem	Unknown Species	—	—	0/59
Timofte et al. (67)	2010	3/17	17.65%	Clinical	The United Kingdom	281.9	Auto	Kpn	—	Drug resistance	3/3
Fuenzalida et al. (68)	2011.05–2013.11	11/279	3.94%	Clinical	The United States	36.39	Biochem	Unknown Species	—	—	—
Langoni et al. (69)	2013	6/4,268	0.14%	—	Brazil	25.6	16S rDNA	Kpn	—	—	—
Sudarwanto et al. (43)	2011.11–2011.12	7/80	8.75%	—	Indonesia	152.6	—	Kpn	PFGE	Drug resistance	8/8
Xie et al. (70)	2013	35/593	5.90%	—	China	153.8	Biochem	Kox	—	—	—
Koovapra et al. (34)	2016	154/340	45.29%	Both	India	468.7	—	Unknown Species	—	Drug resistance	23/291
Levison et al. (71)	2011.04–2012.05	18/936	1.92%	Clinical	Canada	4.168	Biochem	Unknown Species	—	—	—
Rodríguez (72)	2015.09–2015.12	46/142	32.39%	—	Peru	26.06	—	Unknown Species	—	—	—
Diab et al. (49)	2015.09–2015.11	35/154	22.73%	—	Lebanon	661.7	MALDI-TOF	Kpn	PFGE	Drug resistance	36/36
Gao et al. (73)	2014.03–2016.09	426/3,288	12.96%	Clinical	China	153.8	Biochem, 16S rDNA, and Auto	Unknown Species	—	—	—
He et al. (45)	2015	2/65	3.08%	Clinical	China	153.8	MALDI-TOF	Kpn	PFGE	Drug resistance	2/2
Schabauer et al. (74)	2015.10–2016.09	11/3,020	0.36%	—	Austria	109.7	16S rDNA	Kpn	—	—	—
Suleiman et al. (75)	2014.01–2014.07	79/1,648	4.79%	Subclinical	Tanzania	69.43	Biochem	Unknown Species	—	—	—
Tomazi et al. (76)	2014.03–2016.01	110/4,212	2.61%	Clinical	Brazil	25.6	Biochem	Unknown Species	—	—	—
Wang (77)	2017.04	13/57	22.81%	Clinical	China	153.8	Biochem	Kpn	—	—	—
Bach et al. (78)	2017.02–2017.05	6/396	1.52%	—	The United States	36.39	Biochem	Unknown Species	—	—	—
Feng et al. (79)	2017	32/200	16.00%	Clinical	China	153.8	16S rDNA	Unknown Species	—	—	17/32
Hozyen et al. (80)	2017.09–2017.12	7/24	29.17%	Clinical	Egypt	104.7	Biochem	Kpn	—	—	—
Salauddin et al. (36)	2019	30/48	62.50%	Clinical	Bangladesh	1278	Biochem	Unknown Species	—	—	30/30
Zhou et al. (81)	2018.06	13/50	26.00%	Subclinical	China	153.8	Biochem and 16S rDNA	Kpn	—	—	—
Fuenzalida and Ruegg (82)	2016.06–2016.12	54/168	32.14%	Clinical	The United States	36.39	—	Kpn	PFGE	—	—
Griffioen et al. (29)	2017.05–2018.07	1/163	0.61%	Clinical	The Netherlands	509.3	MALDI-TOF	Kpn	—	—	—
Jiang et al. (83)	2019.06–2019.10	2/31	6.45%	Clinical	China	153.8	16S rDNA	Kpn	—	—	2/2
Abboud et al. (84)	2019.03–2019.04	1/90	1.11%	—	Lebanon	661.7	MALDI-TOF	Kox	—	—	0/1
Cheng et al. (85)	2019	206/916	22.49%	Both	China	153.8	Biochem and 16S rDNA	Kpn	—	Virulence	—
Chung et al. (86)	2020.04–2020.09	1/234	0.43%	Both	Australia	3.357	MALDI-TOF	Kpn	—	—	—
Deng et al. (87)	2019.09–2019.12	51/140	36.43%	Clinical	China	153.8	16S rDNA and Auto	Kpn	—	Virulence	51/130
Duse et al. (88)	2013.08–2018.12	22/755	2.91%	Clinical	Sweden	24.76	MALDI-TOF	Unknown Species	—	—	0/22
Enferad and Mahdavi (35)	2018.04–2018.10	80/200	40.00%	—	Iran	52.21	Biochem	Kpn	—	Drug resistance	56/80
Fréchette et al. (89)	2018–2019	56/1,144	4.90%	Clinical	Canada	4.168	MALDI-TOF	Unknown Species	—	—	—
Nobrega et al. (90)	2009.12–2011.07	28/1,547	1.81%	—	Brazil	25.6	Biochem	Kpn	—	Drug resistance	13/81
Taniguchi et al. (8)	2016.08–2017.07	197/1,549	12.72%	—	Japan	345.8	MALDI-TOF	Kpn and Kox	RAPD	Drug resistance	22/197
Tsuka et al. (91)	2012.10–2014.12	97/1,466	6.62%	Clinical	Japan	345.8	Auto	Kpn	PFGE	Drug resistance	—
Yang et al. (92)	2017–2018	59/857	6.88%	Clinical	China	153.8	16S rDNA	Kpn	—	Drug resistance	4/66
Dyson et al. (93)	2011.01–2012.03	20/2,572	0.78%	—	Australia	3.357	Biochem	Unknown Species	—	—	—

^a^No. postive/No. tested: the ratio of *Klebsiella*-positive milk samples to total tested milk samples.

^b^Population density: unit**—**per sq km.

^c^Detection methods: Biochem, biochemical tests; 16S rDNA, 16S rDNA identification; MALDI-TOF, MALDI-TOF MS; Auto, automatic analysis systems.

^d^*Klebsiella* species: Kpn, *Klebsiella pneumoniae*; Kox, *Klebsiella oxytoca*; Koz, *Klebsiella ozaenae*.

^e^Gene detection: molecular characterization of drug resistance genes or virulence genes.

^f^MDR rate: the MDR rate of *Klebsiella* isolates.

^g^Both: studies with both clinical mastitis milk samples and subclinical mastitis milk sample.

Although *Klebsiella* spp. were occasionally detected in milk samples from EU member countries (Table 5), the drug-resistance rate of these bacteria has declined since 2009 (40), and the occurrence of MDR strains is very rare. This may be attributed to the EU's policy of a total ban on the use of antibiotic growth promoters (AGPs) in animal feeding since 2006 (41), which substantially reduced the probability of *Klebsiella* carrying drug-resistance genes in the dairy farm environment. In this meta-analysis, China had the highest number of reports of MDR *Klebsiella* in milk samples, which included seven studies from different regions of the country. In July 2019, Announcement No. 194 was issued by the Chinese Ministry of Agriculture and Rural Affairs to prohibit all antimicrobials from being used as AGPs and was implemented in on July 1, 2020 (42). However, whether this will achieve the desired governance results is pending further investigations in the future.

**Table 5:** Estimated pooled prevalence of *Klebsiella*-positive milk samples by country.

**Country**	*Klebsiella* species^*^(No. studies)	**Continent**	**No. tested**	**No. positive**	**Positive percentage**	**95% CI**
Australia	Kpn (1); Unknown Species (1)	Oceania	2,806	21	0.67%	0.38–1.03%
Austria	Kpn (1)	Europe	3,020	11	0.36%	0.18–0.62%
Bangladesh	Unknown Species (1)	Asia	48	30	62.50%	48.27–75.75%
Belgium	Unknown Species (2)	Europe	30,107	112	0.22%	0.02–0.64%
Brazil	Kpn (3); Kox (1); Koz (1)	South America	10,287	171	2.56%	0.56–5.89%
Canada	Unknown Species (3)	North America	2,278	78	2.90%	1.14–5.39%
China	Kpn (7); Kox (1); Unknown Species (7)	Asia	6,648	873	11.67%	8.00–15.89%
Colombia	Unknown Species (1)	South America	7,954	104	1.31%	1.07–1.57%
Egypt	Kpn (2); Kox (1)	Africa	123	38	30.72%	22.70–39.33%
Estonia	Unknown Species (1)	Europe	4,679	87	1.86%	1.49–2.27%
Ethiopia	Kpn (2); Unknown Species (1)	Africa	383	17	4.13%	2.12–6.68%
France	Unknown Species (1)	Europe	1,770	17	0.96%	0.55–1.47%
India	Unknown Species (1)	Asia	340	154	45.29%	40.03–50.61%
Indonesia	Kpn (1)	Asia	80	7	8.75%	3.39–16.08%
Iran	Kpn (1)	Asia	200	80	40.00%	33.30–46.89%
Japan	Kpn (2); Kox (1)	Asia	3,015	294	9.45%	4.35–16.24%
Korea	Unknown Species (1)	Asia	2,041	107	5.24%	4.32–6.25%
Lebanon	Kpn (1); Kox (1)	Asia	244	36	9.22%	0.00–39.36%
Peru	Unknown Species (1)	South America	142	46	32.39%	24.92–40.34%
Sweden	Unknown Species (1)	Europe	755	22	2.91%	1.82–4.25%
Tanzania	Unknown Species (1)	Africa	1,648	79	4.79%	3.81–5.88%
The Netherlands	Kpn (1); Unknown Species (1)	Europe	303	5	1.52%	0.04–4.41%
The United Kingdom	Kpn (1)	Europe	17	3	17.65%	2.57–39.97%
The United States	Kpn (2); Unknown Species (2)	North America	867	81	14.61%	1.94–35.23%
Uganda	Kox (1)	Africa	97	5	5.15%	1.48–10.61%
Total	—	—	79,852	2,478	7.95%	6.07–10.06%

^*^Klebsiella species: Kpn, *Klebsiella pneumoniae*; Kox, *Klebsiella oxytoca*; Koz, *Klebsiella ozaenae*.

We found that reports of MDR *Klebsiella* spp. in milk samples were also mainly from densely populated developing countries (189/283, 66.78%). Similar to the human carbapenemase-resistant *Klebsiella* species (12), the studies we included suggest that carbapenemase-resistant *Klebsiella* spp. in milk samples are also harbor *bla*_KPC_, *bla*_NDM_ or *bla*_OXA_. KPC-producing *K. pneumoniae* has caused serious nosocomial and community infections worldwide, but there are few reports of their detection in milk samples. Among the articles screened in this study, KPC-producing strains were only reported from bulk tank milk in Indonesia (43). Discovered in 2008 (44), the endemic scope of human NDM-producing *K. pneumoniae* was mainly concentrated in India, Pakistan and Bangladesh. There is also a high prevalence of MDR *K. pneumoniae* in milk samples from some regions of South Asia, but the detection of *bla*_NDM_ was ignored by some local reports (36). Accordingly, we recommend that dairy farms in South Asia promote *bla*_NDM_ detection and strengthen environmental management to take effective measures to constrain the global spread of *bla*_NDM_-harboring strains. Although China is not a major epidemic region for NDM-producing *K. pneumoniae* among humans, the presence of *bla*_NDM − 5_-positive *K. pneumoniae* in both milk and fecal samples was reported in Jiangsu Province. Notably, *bla*_NDM − 5_ plasmids carried by these strains are almost identical to the human *K. pneumoniae* plasmid (pNDM-MGR194) previously reported in India (45), which may be a molecular epidemiological clue of cross-species transmission of *bla*_NDM − 5_ plasmids between humans and cows. Therefore, we need to be alert to the risk of cross-species transmission of the *bla*_NDM − 5_ gene. OXAs commonly hydrolyze isoxazolylpenicillins (oxacillin, cloxacillin, dicloxacillin, etc.), but the hydrolysis of carbapenems by OXA-48 should not be underestimated (46). OXA-48-producing *K. pneumoniae* was first identified in Turkey in 2001 (47), and its presence has been reported in Mediterranean countries (12). In this study, we found that milk-derived OXA-48-producing *K. pneumoniae* also mainly occurred in Mediterranean countries, where their epidemic scope overlaps with that of human strains. According to sampling data from 2008, *bla*_OXA_-harboring *K. oxytoca* strains were detected in milk samples from Egypt (48). Similar reports were subsequently reported in Lebanon, where there was a substantial risk of OXA-48-producing *K. pneumoniae* being transmitted back to humans due to the local practice of selling raw milk (49). In addition, the endemic scope of these strains has been seen in India and other South Asian countries, and milk-derived variants with both *bla*_OXA − 1_ and *bla*_NDM − 5_ genes have been identified in China (45). Therefore, to prevent the emergence of more resistant strains due to the combination of drug-resistance genes, the genetic evolution and integration of *bla*_OXA_ in dairy herds deserves further study.

Among the selected articles, there were only two reports on virulence genes of milk-derived *Klebsiella*, and both of them were from China (Table 4), a region with a high incidence of hypervirulent *K. pneumoniae* (hvKP) infection (13). *Klebsiella* spp. from milk samples also have virulence-related genes associated with siderophore biosynthesis, fimbria proteins, adhesion, and secretion systems, but they are far less abundant than human-derived *K. pneumoniae* strains in terms of gene variety (94). The ferric uptake operon *kfuABC* carried by human-derived *K. pneumoniae* is closely related to colonization and tissue invasion (95). *kfuABC* of the milk-derived strains also has similar functions, and the gene cluster was more prevalent in CM cases than in SCM cases (94), which explained why the prevalence of *Klebsiella* in CM milk samples was significantly higher than that in SCM milk samples. Capsular polysaccharides (CPSs) have been identified as the most important virulence factor of *K. pneumoniae* for assisting bacteria in evading host immune surveillance, and their synthesis is mainly regulated by CPS-regulated genes such as regulator of mucoid phenotype A (*rmpA*) (96, 97). However, *rmpA* was detected only in milk-derived *K. pneumoniae* from China according to this analysis (85). It was found that *K. pneumoniae* was more likely to generate capsules in milk than in LB medium (98), which may indicate that the nutrient-rich environment reduces the dependence of *Klebsiella* capsule synthesis on CPS regulatory genes such as *rmpA*. Despite the extremely low incidence of *rmpA* in milk-derived *Klebsiella* strains, this gene can be transferred with pLVPK-like plasmids to MDR *K. pneumoniae* (especially serotype K47), leading to the emergence of carbapenem-resistant hypervirulent *K. pneumoniae* (CR-hvKP), which has become a more troublesome pathogen of nosocomial infections than MDR KP and hvKP in recent years (99). A study included in our meta-analysis showed that K47 serotype *K. pneumoniae* had been detected in milk samples from Jiangsu, China (92). Therefore, it is necessary to strengthen the monitoring of the genetic evolution of milk-derived *Klebsiella* to prevent the emergence and spread of CR-hvKP in dairy herds.

The advantages of this meta-analysis are its wide geographical coverage, long time span and clear analysis methods, but we have to acknowledge that this study has some limitations. First, the 55 articles in this study were derived from six large databases (three English databases and three Chinese databases), which may have led to the omission of eligible articles from other databases. Due to the constraints of the databases, the majority of the selected articles were in English or Chinese, with only two in other languages (Korean and Spanish), which may have resulted in studies in other languages being omitted. Possibly due to the language, the studies we have included only covered 25 countries. In addition to one case of *K. ozaenae*, only six studies reported *Klebsiella* mastitis caused by *K. oxytoca* with a relatively small sample which may have led to small sample size bias. About half of the included studies did not identify specific *Klebsiella* species, so this meta-analysis mainly focused on *Klebsiella* spp. in dairy milk of cattle with mastitis. Moreover, data was unevenly distributed across countries due to the factors such as research conditions, government attention and trade protection. Unfortunately, we did not find data applicable to this meta-analysis for large milk producing countries such as Russia or New Zealand. However, it is certain that the data cover the characteristics of most dairy farming regions worldwide and can reflect the global distribution and variation trends of *Klebsiella* in milk samples. Second, bovine mastitis generally occurs in multiparous cows (100), but we were unable to extract available data on the parity of cows to find its correlation with the positive rate of *Klebsiella* in milk samples. Third, among the studies collected in this meta-analysis, pathogen identification methods for milk samples included biochemical tests, 16S rDNA identification, MALDI-TOF MS, and automatic analysis systems that were widely adopted to identify the pathogen of the milk samples. It is generally accepted that MALDI-TOF MS or automatic analysis systems are considered to have a lower detection error than conventional biochemical tests (101). Reports from developed economies typically involve 2–3 laboratory tests that provide accurate calibration of pathogen incidence in milk samples. In some developing countries, however, only biochemical tests are usually available, which is likely to result in a small number of missed cases. Therefore, we need to prepare for the worsening outbreaks of *Klebsiella* in dairy herds in some developing countries, and call on local authorities to put more effective measures in place.

## Conclusions

This meta-analysis revealed that the prevalence of *Klebsiella*-positive milk samples and MDR *Klebsiella* spp. are highly correlated with the economic development level and population density of the country or region. These two factors are often prerequisites for banning AGPs and enforcing environmental management. The milk-derived MDR strains from Asia and the Mediterranean region suggest that these strains may have the potential for interspecies transmission between humans and dairy cows, and that MDR strains in Chinese dairy herds may evolve into CR-hvKP. Our meta-analysis provides a reference for the prevention and treatment of bacterial mastitis in dairy cows and the blocking of the global transmission of zoonotic pathogens.

## Data availability statement

The original contributions presented in the study are included in the article/Supplementary material, further inquiries can be directed to the corresponding authors.

## Author contributions

RC, RD, and QG conceptualized the study, with funding provided by RC and were responsible for the revision. JS, WX, QW, JY, TT, QY, MZ, GG, JL, ND, FL, and KS performed data extraction. JS and WX established the database and wrote the original draft of the article. QW carried out the data analysis. All authors contributed to the manuscript editing and approved the final manuscript.
