# Supplementary material for: Prevalence and risk factors of Klebsiella spp. in milk samples from dairy cows with mastitis—A global systematic review
Source: Front Vet Sci. 2023 Mar 22;10:1143257. doi: 10.3389/fvets.2023.1143257 (PMC10073557; doi:10.3389/fvets.2023.1143257)
Supplement: Supplementary file 1 [file Data_Sheet_1.docx]

***Supplementary Material***

**Prevalence and risk factors of *Klebsiella* spp. in milk samples from dairy cows with mastitis—A global systematic review**

**Jinming Song^†^, Wentao Xiang^†^, Qi Wang^†^, Jiying Yin, Tian Tian, Qizhu Yang, Meng Zhang, Guiyang Ge, Jianming Li, Naichao Diao, Fei Liu, Kun Shi, Ruopeng Cai^*^, Rui Du^*^, Qinglong Gong^*^**

*** Correspondence:** Ruopeng Cai: [saycall850@vip.qq.com](mailto:saycall850@vip.qq.com); Rui Du: [durui197101@sina.com](mailto:durui197101@sina.com); Qinglong Gong: [gongqinglong1001@163.com](mailto:gongqinglong1001@163.com)

**Equal contributions:** † These authors have contributed equally to this work.

1. **Supplementary Figures**

**
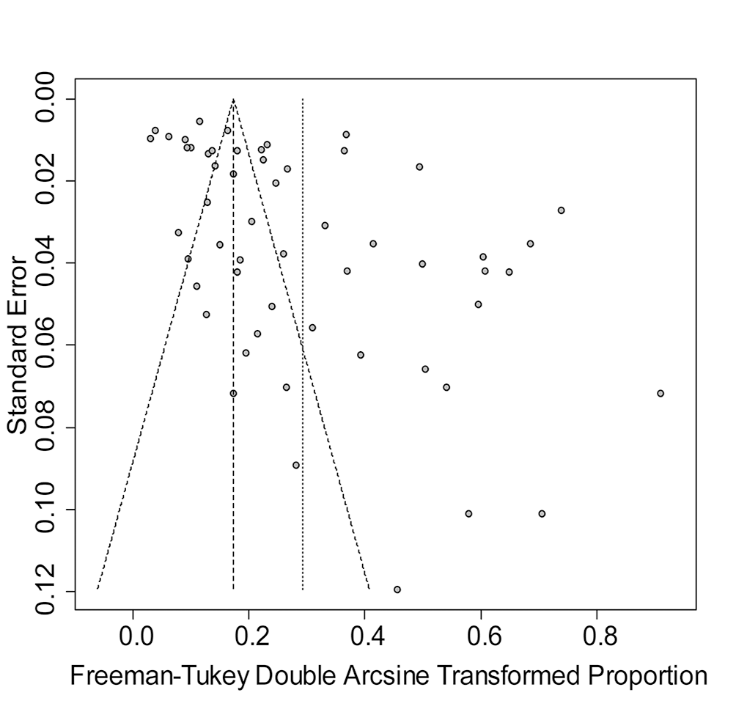
**

**Supplementary Figure 1.** Funnel plot with pseudo 95% confidence limits intervals for the examination of publication bias of sampling years.

**
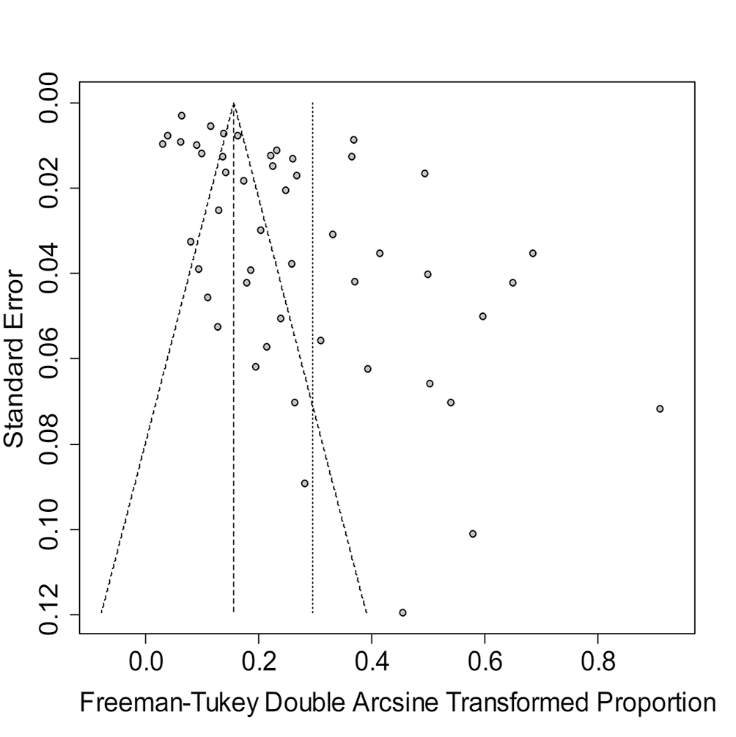
Supplementary Figure 2.** Funnel plot with pseudo 95% confidence limits intervals for the examination of publication bias of detection methods.

**
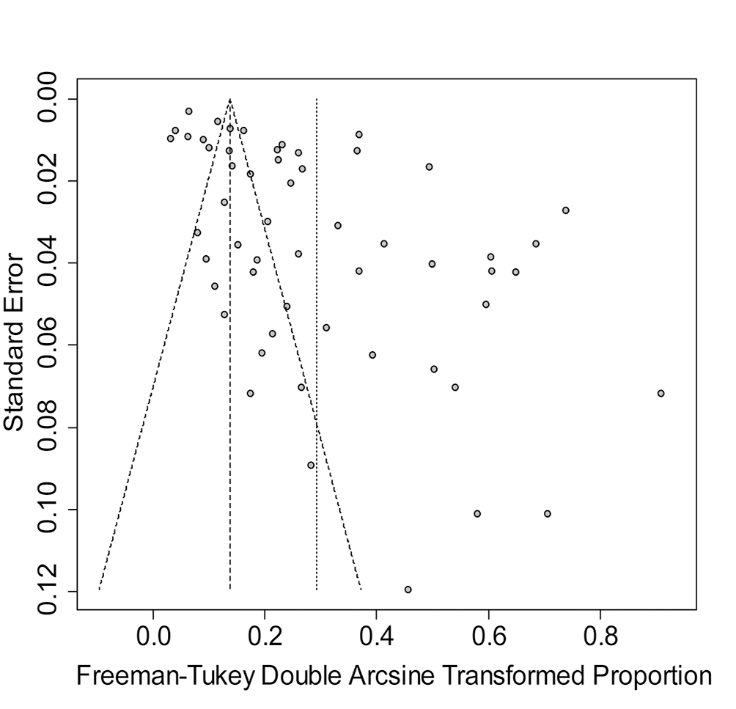
Supplementary Figure 3.** Funnel plot with pseudo 95% confidence limits intervals for the examination of publication bias of continents.

**
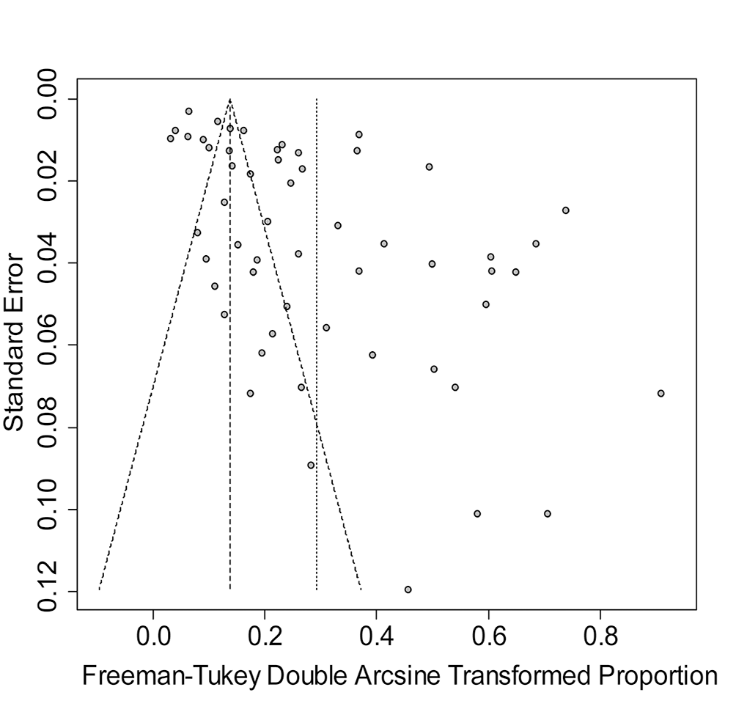
Supplementary Figure 4.** Funnel plot with pseudo 95% confidence limits intervals for the examination of publication bias of economic development level.

**
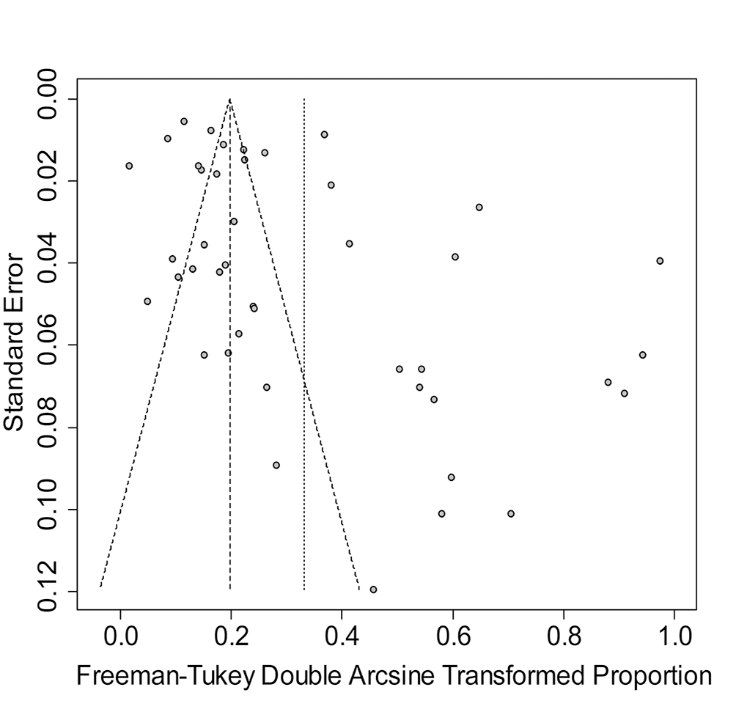
Supplementary Figure 5.** Funnel plot with pseudo 95% confidence limits intervals for the examination of publication bias of mastitis type.

**
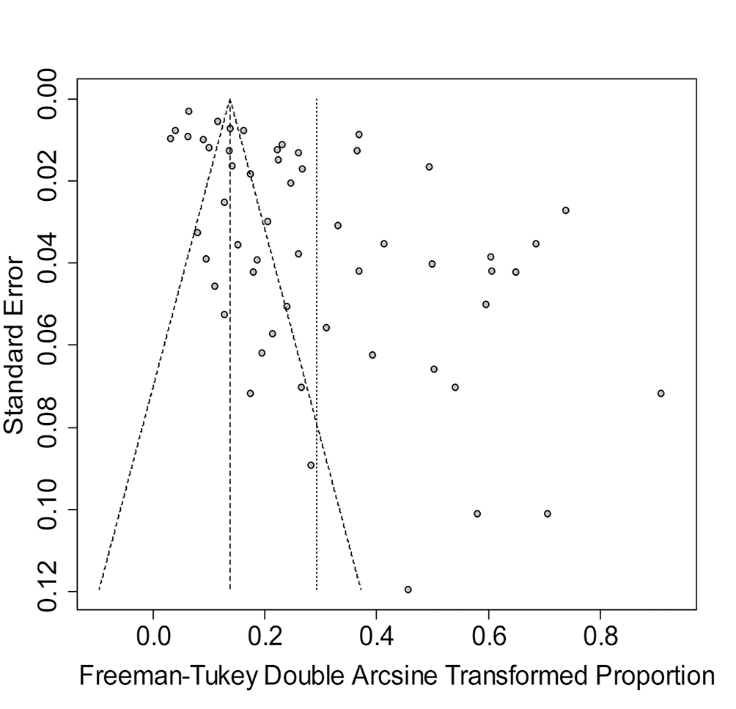
Supplementary Figure 6.** Funnel plot with pseudo 95% confidence limits intervals for the examination of publication bias of population density.

**
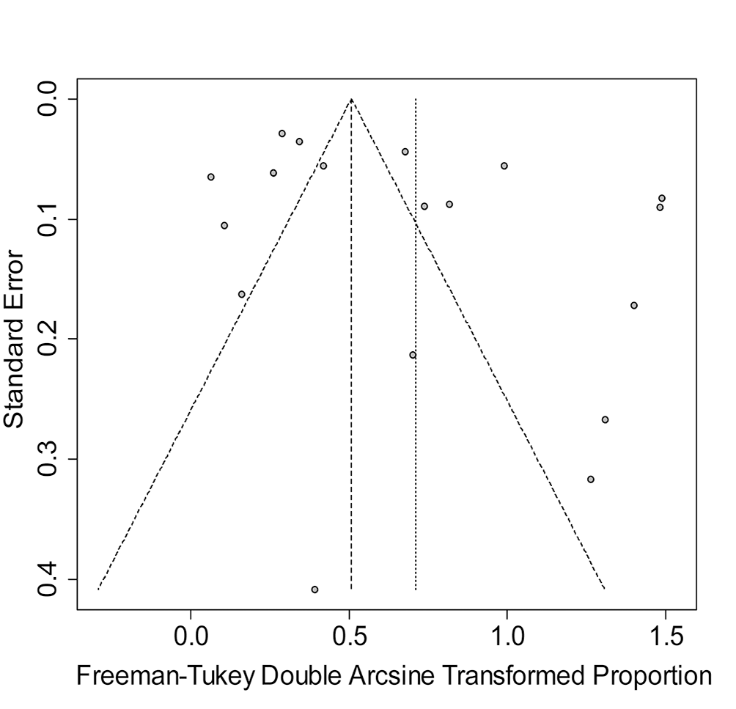
Supplementary Figure 7.** Funnel plot with pseudo 95% confidence limits intervals for the examination of publication bias of the MDR rate.

**
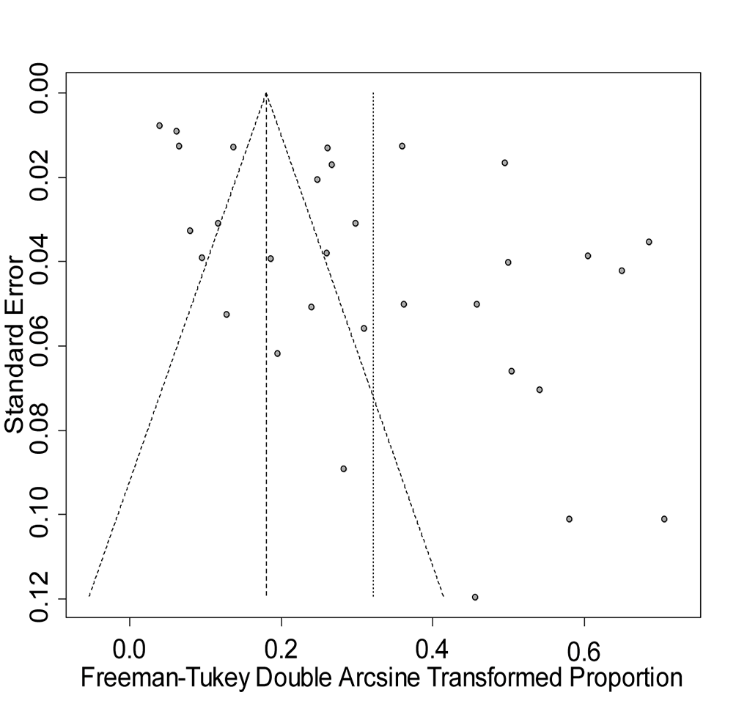
Supplementary Figure 8.** Funnel plot with pseudo 95% confidence limits intervals for the examination of publication bias of *Klebsiella* species.

1. **Supplementary Tables**

**Supplementary Table 1.** The code in R for meta-analysis.

| Logarithmic conversion (PLN) | rate<-transform [m1, log=log(event/n)];  shapiro.test(rate$log) |
| --- | --- |
| Logit transformation (PLOGIT) | rate<-transform{m1, logit=log[(event/n)/(1-event/n)]};  shapiro.test(rate$logit) |
| Arcsine transformation (PAS) | rate<-transform{m1, arcsin.size=asin[sqrt(event/(n+1))]};  shapiro.test(rate$arcsin) |
| Double-arcsine transformation (PFT) | rate<-transform{m1,darcsin=0.5*[asin(sqrt(event/(n+1)))+asin((sqrt(event+1)/(n+1)))]};  shapiro.test(rate$darcsin) |
| No transformation (PRAW) | rate<-transform[m1, r= event/n];  shapiro.test(rate$r) |
| Forest plots | forest [meta1, xlim=c(-0.4, 1)] |
| Funnel chart | funnel (meta1) |
| Egger's test | metabias (meta1, method="linreg") |
| The sensitivity analysis | metainf (meta1, pooled = "random") forest (metainf (meta1, pooled = "random"), xlim=c(0, 0.2)) |
| Subgroup analysis | meta1<-metaprop(event, n, study, data=rate, sm="PLN", incr=0.5, allincr=TRUE, addincr=FALSE, title="", byvar= subgroup title, print.byvar=TRUE) |
| Meta-regression analysis | metareg (meta1, ~covariate title) |

**Supplementary Table 2.** Included studies and quality scores.

|  | Reference ID | No.  tested | No.  positive | Prevalence | Random sampling or not | Sampled method detailly or not | Sample time clearly or not | Detection method clearly or not | Four or more risk factors or not | Score |
| --- | --- | --- | --- | --- | --- | --- | --- | --- | --- | --- |
| 1 | Munoz et al. (2007) | 24 | 10 | 41.67% | N^1^ | Y^2^ | Y | Y | Y | 4 |
| 2 | Liu (2008) | 120 | 1 | 0.83% | N | Y | Y | N | Y | 3 |
| 3 | Botrel et al. (2010) | 1,770 | 17 | 0.96% | N | Y | Y | Y | Y | 4 |
| 4 | Mekibib et al. (2010) | 161 | 5 | 3.11% | N | Y | Y | Y | Y | 4 |
| 5 | Zanella et al. (2010) | 260 | 27 | 10.38% | N | Y | N | Y | Y | 3 |
| 6 | Ahmed and Shimamoto (2011) | 99 | 31 | 31.31% | N | N | Y | Y | Y | 3 |
| 7 | Guo (2011) | 141 | 18 | 12.77% | N | Y | Y | Y | Y | 4 |
| 8 | Kalmus et al. (2011) | 4,679 | 87 | 1.86% | N | Y | N | Y | Y | 3 |
| 9 | Kamphuis et al. (2011) | 140 | 4 | 2.86% | Y | Y | N | N | Y | 3 |
| 10 | Verbist et al. (2011) | 2,644 | 2 | 0.08% | Y | Y | Y | Y | Y | 5 |
| 11 | Abera et al. (2012) | 48 | 1 | 2.08% | Y | Y | Y | Y | Y | 5 |
| 12 | Ba and Li (2012) | 64 | 9 | 14.06% | Y | Y | Y | Y | Y | 5 |
| 13 | Haftu et al. (2012) | 174 | 11 | 6.32% | Y | Y | Y | Y | Y | 5 |
| 14 | Liu (2012) | 50 | 3 | 6.00% | N | Y | Y | Y | Y | 4 |
| 15 | Wang et al. (2012) | 76 | 3 | 3.95% | N | Y | N | Y | Y | 3 |
| 16 | Kateete et al. (2013) | 97 | 5 | 5.15% | N | Y | N | Y | Y | 3 |
| 17 | Nam et al. (2013) | 2,041 | 107 | 5.24% | N | Y | N | Y | Y | 3 |
| 18 | Thompson et al. (2013) | 198 | 4 | 2.02% | N | N | Y | Y | Y | 3 |
| 19 | Ramírez et al. (2014) | 7,954 | 104 | 1.31% | N | Y | Y | Y | Y | 4 |
| 20 | Supre et al. (2014) | 27463 | 110 | 0.40% | N | Y | N | Y | Y | 3 |

(*Continued*)

**Supplementary Table 2.** Continued

|  | Reference ID | No.  tested | No.  positive | Prevalence | Random sampling or not | Sampled method detailly or not | Sample time clearly or not | Detection method clearly or not | Four or more risk factors or not | Score |
| --- | --- | --- | --- | --- | --- | --- | --- | --- | --- | --- |
| 21 | Timofte et al. (2014) | 17 | 3 | 17.65% | N | Y | N | Y | Y | 3 |
| 22 | Fuenzalida et al. (2015) | 279 | 11 | 3.94% | N | Y | Y | Y | Y | 4 |
| 23 | Langoni et al. (2015) | 4,268 | 6 | 0.14% | N | Y | Y | N | Y | 3 |
| 24 | Sudarwanto et al. (2015) | 80 | 7 | 8.75% | Y | Y | Y | Y | Y | 5 |
| 25 | Xie et al. (2015) | 593 | 35 | 5.90% | N | Y | Y | Y | Y | 4 |
| 26 | Koovapra et al. (2016) | 340 | 154 | 45.29% | N | Y | Y | N | Y | 3 |
| 27 | Levison et al. (2016) | 936 | 18 | 1.92% | N | Y | Y | Y | Y | 4 |
| 28 | Rodríguez (2016) | 142 | 46 | 32.39% | N | Y | Y | Y | Y | 4 |
| 29 | Diab et al. (2017) | 154 | 35 | 22.73% | N | Y | Y | Y | Y | 4 |
| 30 | Gao et al. (2017) | 3,288 | 426 | 12.96% | N | Y | Y | Y | Y | 4 |
| 31 | He et al. (2017) | 65 | 2 | 3.08% | N | Y | Y | Y | Y | 4 |
| 32 | Schabauer et al. (2018) | 3,020 | 11 | 0.36% | N | Y | Y | Y | Y | 4 |
| 33 | Suleiman et al. (2018) | 1,648 | 79 | 4.79% | N | Y | Y | Y | Y | 4 |
| 34 | Tomazi et al. (2018) | 4,212 | 110 | 2.61% | N | Y | Y | Y | Y | 4 |
| 35 | Wang (2018) | 57 | 13 | 22.81% | N | Y | Y | Y | Y | 4 |
| 36 | Bach et al. (2019) | 396 | 6 | 1.52% | N | Y | Y | Y | Y | 4 |
| 37 | Feng et al. (2019) | 200 | 32 | 16.00% | N | Y | Y | Y | Y | 4 |
| 38 | Hozyen et al. (2019) | 24 | 7 | 29.17% | N | Y | Y | Y | Y | 4 |
| 39 | Salauddin et al. (2019) | 48 | 30 | 62.50% | N | Y | N | N | Y | 2 |
| 40 | Zhou et al. (2019) | 50 | 13 | 26.00% | N | Y | Y | Y | Y | 4 |
| 41 | Fuenzalida and Ruegg (2020) | 168 | 54 | 32.14% | Y | Y | Y | Y | Y | 5 |
| 42 | Griffioen et al. (2020) | 163 | 1 | 0.61% | N | Y | Y | Y | Y | 4 |

(*Continued*)

**Supplementary Table 2.** Continued

|  | Reference ID | No.  tested | No.  positive | Prevalence | Random sampling or not | Sampled method detailly or not | Sample time clearly or not | Detection method clearly or not | Four or more risk factors or not | Score |
| --- | --- | --- | --- | --- | --- | --- | --- | --- | --- | --- |
| 43 | Jiang et al. (2020) | 31 | 2 | 6.45% | N | Y | Y | Y | Y | 4 |
| 44 | Abboud et al. (2021) | 90 | 1 | 1.11% | N | Y | Y | Y | Y | 4 |
| 45 | Cheng et al. (2021) | 916 | 206 | 22.49% | N | Y | Y | Y | Y | 4 |
| 46 | Chung et al. (2021) | 234 | 1 | 0.43% | N | Y | Y | Y | Y | 4 |
| 47 | Deng et al. (2021) | 140 | 51 | 36.43% | N | Y | Y | Y | Y | 4 |
| 48 | Duse et al. (2021) | 755 | 22 | 2.91% | N | Y | Y | Y | Y | 4 |
| 49 | Enferad and Mahdavi (2021) | 200 | 80 | 40.00% | Y | Y | Y | Y | Y | 5 |
| 50 | Fréchette et al. (2021) | 1,144 | 56 | 4.90% | N | Y | Y | Y | Y | 4 |
| 51 | Nobrega et al. (2021) | 1,547 | 28 | 1.81% | N | Y | Y | Y | Y | 4 |
| 52 | Taniguchi et al. (2021) | 1,549 | 197 | 12.72% | N | Y | Y | Y | Y | 4 |
| 53 | Tsuka et al. (2021) | 1,466 | 97 | 6.62% | N | Y | Y | Y | Y | 4 |
| 54 | Yang et al. (2021) | 857 | 59 | 6.88% | Y | Y | Y | Y | Y | 5 |
| 55 | Dyson et al. (2022) | 2,572 | 20 | 0.78% | N | Y | Y | Y | Y | 4 |

*^1^N: no.*

*^2^Y: yes.*

**References of studies included in the analysis**:

1. Munoz, M.A., Welcome, F.L., Schukken, Y.H., Zadoks, R.N., 2007. Molecular epidemiology of two *Klebsiella pneumoniae* mastitis outbreaks on a dairy farm in New York State. J. Clin. Microbiol. 45(12):3964-3971. <https://doi.org/10.1128/JCM.00795-07>.
2. Liu, X., 2008. Etiology investigation and drug sensitive test of the pathogenic bacteria of cow mastitis in Baotou region (in Chinese). MS Thesis. Northwest A&F Univ., the People’s Republic of China.
3. Botrel, M.A., Haenni, M., Morignat, E., Sulpice, P., Madec, J.Y., Calavas, D., 2010. Distribution and antimicrobial resistance of clinical and subclinical mastitis pathogens in dairy cows in Rhône-Alpes, France. Foodborne Pathog. Dis. 7(5):479-487. <https://doi.org/10.1089/fpd.2009.0425>.
4. Mekibib, B., Furgasa, M., Abunna, F., Megersa, B., Regassa. A., 2010. Bovine mastitis: Prevalence, risk factors and major pathogens in dairy farms of Holeta Town, Central Ethiopia. Vet. world 3(9):397-403. [http://doi.org/10.5455/vetworld.2010.397-403](http://dx.doi.org/10.5455/vetworld.2010.397-403).
5. Zanella, G., Mikcha, J., Bando, E., Siqueira, V., Machinski M.Jr., 2010. Occurrence and antibiotic resistance of coliform bacteria and antimicrobial residues in pasteurized cow's milk from Brazil. J. Food Prot. 73(9):1684-1687. <https://doi.org/10.4315/0362-028x-73.9.1684>.
6. Ahmed, A.M., Shimamoto., T., 2011. Molecular characterization of antimicrobial resistance in Gram-negative bacteria isolated from bovine mastitis in Egypt. Microbiol. Immunol. 55(5):318-327. <https://doi.org/10.1111/j.1348-0421.2011.00323.x>.
7. Guo, C.Y., 2011. Studies on the cow mastitis and environment pathogenic bacteria of an intensive cow farm (in Chinese). MS Thesis. Inner Mongolia Agricultural Univ., the People’s Republic of China.
8. Kalmus, P., Aasmäe, B., Kärssin, A., Orro, T., Kask., K., 2011. Udder pathogens and their resistance to antimicrobial agents in dairy cows in Estonia. Acta. Vet. Scand. 53(1):1-7. <https://doi.org/10.1186/1751-0147-53-4>.
9. Kamphuis, C., Mollenhorst, H., Hogeveen., H., 2011. Sensor measurements revealed: Predicting the Gram-status of clinical mastitis causal pathogens. Comput. Electron. Agric. 77(1):86-94. <https://doi.org/10.1016/j.compag.2011.03.012>.
10. Verbist, B., Piessens, V., Van Nuffel, A., De Vuyst, L., Heyndrickx, M., Herman, L., Van Coillie, E., De Vliegher., S., 2011. Sources other than unused sawdust can introduce *Klebsiella pneumoniae* into dairy herds. J. Dairy Sci. 94(6):2832-2839. <https://doi.org/10.3168/jds.2010-3700>.
11. Abera, M., Habte, T., Aragaw, K., Asmare, K., Sheferaw., D., 2012. Major causes of mastitis and associated risk factors in smallholder dairy farms in and around Hawassa, Southern Ethiopia. Trop. Anim. Health Prod. 44(6):1175-1179. <https://doi.org/10.1007/s11250-011-0055-3>.
12. Ba, K.M., Li, X.Y., 2012. Isolation and identification of mastitis pathogenic bacteria and their pathogenicity and drug sensitivity tests in dairy cows in the Hohhot area (in Chinese). Mod. Anim. Husb. 2012(12):15-18. [https://doi.org/10.14070/j.cnki.15-1150.2012.12.013](https://kns.cnki.net/kcms/detail/detail.aspx?FileName=DAYZ201212008&DbName=CJFQ2012).
13. Haftu, R., Taddele, H., Gugsa, G., Kalayou, S., 2012. Prevalence, bacterial causes, and antimicrobial susceptibility profile of mastitis isolates from cows in large-scale dairy farms of Northern Ethiopia. Trop. Anim. Health Prod. 44(7):1765-1771. <https://doi.org/10.1007/s11250-012-0135-z>.
14. Liu, M.C. 2012. The investigation and treatment with Chinese herbal medicine of subclinical mastitis of dairy cattle in Liuzhou (in Chinese). MS Thesis. Nanjing Agricultural Univ., the People’s Republic of China.
15. Wang, X.Y., Li, H.S., Li, J.X., Wang, X.H., Meng, J.R., Yang, F., Yang, Z.Q., 2012. Identification of bacteria isolated from dairy cows with clinical type mastitis and analysis of drug resistance (in Chinese). China Anim. Husb. Vet. Med. 39(07):195-198.
16. Kateete, D.P., Kabugo, U., Baluku, H., Nyakarahuka, L., Kyobe, S., Okee, M., Najjuka, C.F., Joloba, M.L., 2013. Prevalence and antimicrobial susceptibility patterns of bacteria from milkmen and cows with clinical mastitis in and around Kampala, Uganda. PLoS One 8(5):e63413. <https://doi.org/10.1371/journal.pone.0063413>.
17. Nam, H.M., Lim, S.K., Jang, G.C., Joung, D.Y., Kim, H.j. Lee, C.S., Jung, S.C., 2013. Culture results from quarter milk samples submitted to veterinary diagnostic laboratories during January~November 2012 in Korea. Prev. Vet. Med. 37(3):111-119. <https://doi.org/10.13041/jpvm.2013.37.3.111>.
18. Thompson-Crispi, K.A., Miglior, F., Mallard, B.A., 2013. Incidence rates of clinical mastitis among Canadian Holsteins classified as high, average, or low immune responders. Clin. Vaccine Immunol. 20(1):106-112. <https://doi.org/10.1128/CVI.00494-12>.
19. Ramirez, N. F., Keefe, G., Dohoo, I., Sanchez, J., Arroyave, O., Ceron, J., Jaramillo, M., Palacio. L.G., 2014. Herd- and cow-level risk factors associated with subclinical mastitis in dairy farms from the high plains of the northern Antioquia, Colombia. J. Dairy Sci. 97(7):4141-4150. <https://doi.org/10.3168/jds.2013-6815>.
20. Supre, K., Lommelen, K., De Meulemeester, L., 2014. Antimicrobial susceptibility and distribution of inhibition zone diameters of bovine mastitis pathogens in Flanders, Belgium. Vet. Microbiol. 171(3-4):374-381. <https://doi.org/10.1016/j.vetmic.2014.02.045>.
21. Timofte, D., Maciuca, I.E., Evans, N.J., Williams, H., Wattret, A., Fick, J.C., Williams, N.J., 2014. Detection and molecular characterization of *Escherichia coli* CTX-M-15 and *Klebsiella pneumoniae* SHV-12 beta-lactamases from bovine mastitis isolates in the United Kingdom. Antimicrob. Agents Chemother. 58(2):789-794. <https://doi.org/10.1128/AAC.00752-13>.
22. Fuenzalida, M.J., Fricke, P.M., Ruegg. P.L., 2015. The association between occurrence and severity of subclinical and clinical mastitis on pregnancies per artificial insemination at first service of Holstein cows. J. Dairy Sci. 98(6):3791-3805. <https://doi.org/10.3168/jds.2014-8997>.
23. Langoni, H., Guiduce, M.V.S., Nóbrega, D.B., da Silva, R.C., Richini-Pereira, V.B. Salina, A., Guimarães, F.F., 2015. Research of *Klebsiella pneumoniae* in dairy herds. Pesq. Vet. Bras. 35(1):9-12. <https://doi.org/10.1590/S0100-736X2015000100003>.
24. Sudarwanto, M., Akineden, O., Odenthal, S., Gross, M., Usleber, E., 2015. Extended-spectrum beta-lactamase (ESBL)-producing *Klebsiella pneumoniae* in bulk tank milk from dairy farms in Indonesia. Foodborne Pathog. Dis. 12(7):585-590. <https://doi.org/10.1089/fpd.2014.1895>.
25. Xie, L.H. Ji, W., Liu, J.H., Gao, C., Qi, R.B., Han, C.L., Zhao, X.H., Zheng, C., Zhang, N.N., 2015. Study on the status of endogenous β-lactamase residues in raw cow's milk in Beijing (in Chinese). China Dairy 2015(02):51-56. <https://doi.org/10.16172/j.cnki.114768.2015.02.021>.
26. Koovapra, S., Bandyopadhyay, S., Das, G., Bhattacharyya, D., Banerjee, J., Mahanti, A., Samanta, I., Nanda, P.K., Kumar, A., Mukherjee, R., Dimri, U., Singh, R.K., 2016. Molecular signature of extended spectrum beta-lactamase producing *Klebsiella pneumoniae* isolated from bovine milk in eastern and north-eastern India. Infect. Genet. Evol. 44:395-402. <https://doi.org/10.1016/j.meegid.2016.07.032>.
27. Levison, L.J., Miller-Cushon, E.K., Tucker, A.L., Bergeron, R., Leslie, K.E. Barkema, H.W., DeVries, T.J., 2016. Incidence rate of pathogen-specific clinical mastitis on conventional and organic Canadian dairy farms. J. Dairy Sci. 99(2):1341-1350. <https://doi.org/10.3168/jds.2015-9809>.
28. Rodríguez Pérez, R.O., 2016. Frequency and antimicrobial susceptibility of mastitis-causing bacteria in cattle from a Conache dairy of the Laredo-Trujillo district 2015 [in Spanish]. MS Thesis. Trujillo National Univ., Spain.
29. Diab, M., Hamze, M., Bonnet, R., Saras, E., Madec, J.Y., Haenni, M., 2017. OXA-48 and CTX-M-15 extended-spectrum beta-lactamases in raw milk in Lebanon: epidemic spread of dominant *Klebsiella pneumoniae* clones. J. Med. Microbiol. 66(11):1688-1691. <https://doi.org/10.1099/jmm.0.000620>.
30. Gao, J., Barkema, H.W., Zhang, L., Liu, G., Deng, Z., Cai, L., Shan, R., Zhang, S., Zou, J., Kastelic, J.P., Han, B., 2017. Incidence of clinical mastitis and distribution of pathogens on large Chinese dairy farms. J. Dairy Sci. 100(6):4797-4806. <https://doi.org/10.3168/jds.2016-12334>.
31. He, T., Wei, R., Zhang, L., Sun, L., Pang, M., Wang, R., Wang, Y., 2017. Characterization of NDM-5-positive extensively resistant *Escherichia coli* isolates from dairy cows. Vet. Microbiol. 207:153-158. <https://doi.org/10.1016/j.vetmic.2017.06.010>.
32. Schabauer, A., Pinior, B., Gruber, C.M., Firth, C.L., Kasbohrer, A., Wagner, M., Rychli, K., Obritzhauser, W., 2018. The relationship between clinical signs and microbiological species, spa type, and antimicrobial resistance in bovine mastitis cases in Austria. Vet. Microbiol. 227:52-60. <https://doi.org/10.1016/j.vetmic.2018.10.024>.
33. Suleiman, T.S., Karimuribo, E.D., Mdegela, R.H., 2018. Prevalence of bovine subclinical mastitis and antibiotic susceptibility patterns of major mastitis pathogens isolated in Unguja island of Zanzibar, Tanzania. Trop. Anim. Health Prod. 50(2):259-266. <https://doi.org/10.1007/s11250-017-1424-3>.
34. Tomazi, T., Ferreira, G.C., Orsi, A.M., Goncalves, J.L., Ospina, P.A., Nydam, D.V., Moroni, P., Dos Santos, M.V., 2018. Association of herd-level risk factors and incidence rate of clinical mastitis in 20 Brazilian dairy herds. Prev. Vet. Med. 161:9-18. <https://doi.org/10.1016/j.prevetmed.2018.10.007>.
35. Wang, J., 2018. Investigation on the causes of clinical mastitis in bovine, isolation, identification and drug sensitivity test (in Chinese). MS Thesis. Northwest A&F Univ., the People’s Republic of China.
36. Bach, K.D., Sipka, A., McArt. J.A.A., 2019. Case study: evaluating quarter and composite milk sampling for detection of subclinical intramammary infections in dairy cattle. Prev. Vet. Med. 163:51-57. <https://doi.org/10.1016/j.prevetmed.2018.12.013>.
37. Feng, X.H., Du, L., Wang, L.F., Zhang, S.F., Song, J., Hu, Z.G., 2019. Isolation and identification of *Klebsiella* and analysis of drug resistance from mastitis dairy cows in Shanghai and Hebei in 2017 (in Chinese). Ani. Hus. Feed Sci. 40(01):108-112. <https://doi.org/10.16003/j.cnki.issn1672-5190.2019.01.029>.
38. Hozyen, H.F., Ibrahim, E.S., Khairy, E.A., El-Dek, S.I., 2019. Enhanced antibacterial activity of capped zinc oxide nanoparticles: A step towards the control of clinical bovine mastitis. Vet. World 12(8):1225-1232. [https://dx.doi.org/10.14202/vetworld.2019.1225-1232](https://dx.doi.org/10.14202%2Fvetworld.2019.1225-1232).
39. Salauddin, M., Akter, M.R., Hossain, M.K., Rahman, M.M., 2019. Isolation of multi-drug resistant *Klebsiella* sp. from bovine mastitis samples in Rangpur, Bangladesh. J. Adv. Vet. Anim. Res. 6(3):362-365. [https://dx.doi.org/10.5455/javar.2019.f355](https://dx.doi.org/10.5455%2Fjavar.2019.f355).
40. Zhou, M.X., Zuo, X.X., Xv, Y., Bao, X., Zhang, J.Q., Yang, Z.P., Zhu, G.Q., Lu, Y., 2019. Isolation, identification, drug resistance, and conserved antigen analysis of mastitis pathogenic bacteria from cows in a dairy farm in Jiangsu, China (in Chinese). J. Yangzhou Univ. 40(06):54-60. <https://doi.org/10.16872/j.cnki.1671-4652.2019.06.009>.
41. Fuenzalida, M.J., Ruegg. P.L., 2020. Molecular epidemiology of nonsevere clinical mastitis caused by *Klebsiella pneumoniae* occurring in cows on 2 Wisconsin dairy farms. J. Dairy Sci. 103(4):3479-3492. <https://doi.org/10.3168/jds.2019-17464>.
42. Griffioen, K., Cornelissen, J., Heuvelink, A., Adusei, D., Mevius, D., Jan van der Wal, F., & Health4Food-Dutch Mastitis Diagnostics, 2020. Development and evaluation of 4 loop-mediated isothermal amplification assays to detect mastitis-causing bacteria in bovine milk samples. J. Dairy Sci. 103(9):8407-8420. <https://doi.org/10.3168/jds.2019-18035>.
43. Jiang, L.Z., Xv, Y., Lin, Z.P., Ma, F., Xing, G.D., Zhou, M.X., Lu, Y., 2020. Isolation, identification, and drug resistance of recalcitrant pathogens of mastitis in a dairy farm in northern Jiangsu province (in Chinese). Acta. Agric. Jiangxi 32(02):115-119. <https://doi.org/10.19386/j.cnki.jxnyxb.2020.02.21>.
44. Abboud, Z., Galuppo, L., Tolone, M., Vitale, M., Puleio, R., Osman, M., Loria, G.R., Hamze, M., 2021. Molecular characterization of antimicrobial resistance and virulence genes of bacterial pathogens from bovine and caprine mastitis in Northern Lebanon. Microorganisms 9(6). <https://doi.org/10.3390/microorganisms9061148>.
45. Cheng, J., Zhou, M., Nobrega, D.B., Cao, Z., Yang, J., Zhu, C., Han, B., Gao, J., 2021. Virulence profiles of *Klebsiella pneumoniae* isolated from 2 large dairy farms in China. J. Dairy Sci. 104(8):9027-9036. <https://doi.org/10.3168/jds.2020-20042>.
46. Chung, L.K., Sahibzada, S., Annandale, H.C., Robertson, I.D., Waichigo, F.W., Tufail, M.S. Aleri, J.A., 2021. Bacterial pathogens associated with clinical and subclinical mastitis in a Mediterranean pasture-based dairy production system of Australia. Res. Vet. Sci. 141:103-109. <https://doi.org/10.1016/j.rvsc.2021.10.005>.
47. Deng, B., Wang, X.X., Liu, Y., Sun, X.H., Han, Y.Y., Feng. D.S., 2021. Analysis of virulence genes and drug resistance of *Klebsiella pneumoniae* in raw milk in Shanghai (in Chinese). China Dairy Cattle 2021(06):35-40. <https://doi.org/10.19305/j.cnki.11-3009/s.2021.06.009>.
48. Duse, A., Persson-Waller, K., Pedersen, K., 2021. Microbial aetiology, antibiotic susceptibility and pathogen-specific risk factors for udder pathogens from clinical mastitis in dairy cows. Animals 11(7). <https://doi.org/10.3390/ani11072113>.
49. Enferad, E., Mahdavi S., 2021. Antibiotic resistance pattern and frequency of some beta lactamase genes in *Klebsiella pneumoniae* isolated from raw milk samples in Iran. J. Hellenic Vet. Med. Soc. 71(4). <https://doi.org/10.12681/jhvms.25925>.
50. Frechette, A., Fecteau, G., Cote, C., Dufour, S., 2021. Clinical mastitis incidence in dairy cows housed on recycled manure solids bedding: a Canadian cohort study. Front. Vet. Sci. 8:742868. <https://doi.org/10.3389/fvets.2021.742868>.
51. Nobrega, D.B., Calarga, A.P., Nascimento, L.C., Chande Vasconcelos, C.G., de Lima, E.M., Langoni, H., Brocchi, M., 2021. Molecular characterization of antimicrobial resistance in *Klebsiella pneumoniae* isolated from Brazilian dairy herds. J. Dairy Sci. 104(6):7210-7224. <https://doi.org/10.3168/jds.2020-19569>.
52. Taniguchi, T., Latt, K.M., Tarigan, E., Yano, F., Sato, H., Minamino, T., Misawa, N., 2021. A 1-Year Investigation of Extended-Spectrum Beta-Lactamase-producing *Escherichia coli* and *Klebsiella pneumoniae* isolated from bovine mastitis at a large-scale dairy farm in Japan. Microb. Drug Resist. 27(10):1450-1454. <https://doi.org/10.1089/mdr.2020.0481>.
53. Tsuka, T., Ozaki, H., Saito, D., Murase, T., Okamoto, Y., Azuma, K., Osaki, T., Ito, N., Murahata, Y., Imagawa, T., 2021. Genetic characterization of CTX-M-2-producing *Klebsiella pneumoniae* and *Klebsiella oxytoca* associated with bovine mastitis in Japan. Front. Vet. Sci. 8:659222. <https://doi.org/10.3389/fvets.2021.659222>.
54. Yang, Y., Peng, Y., Jiang, J., Gong, Z., Zhu, H., Wang, K., Zhou, Q., Tian, Y., Qin, A., Yang, Z., Shang. S., 2021. Isolation and characterization of multidrug-resistant *Klebsiella pneumoniae* from raw cow milk in Jiangsu and Shandong provinces, China. Transbound. Emerg. Dis. 68(3):1033-1039. <https://doi.org/10.1111/tbed.13787>.
55. Dyson, R., Charman, N., Hodge, A., Rowe, S.M., Taylor. L.F., 2022. A survey of mastitis pathogens including antimicrobial susceptibility in southeastern Australian dairy herds. J. Dairy Sci. 105(2):1504-1518. <https://doi.org/10.3168/jds.2021-20955>.

**Supplementary Table 3.** Egger’s test for publication bias.

| slope | bias | se.bias | t | df | *p*-value |
| --- | --- | --- | --- | --- | --- |
| 0.06941746 | 7.44378025 | 1.41604355 | 5.2567 | 53 | 2.672e-06 |

**Supplementary Table 4.** Characteristics of the included studies by *Klebsiella* species.

|  | Reference ID | Sampling time | No. positive/  No. tested^1^ | Prevalence | Mastitis  type | Country | Population  Density^2^ | Detection methods^3^ | Genotyping methods | Gene detection^4^ | MDR rate^5^ |
| --- | --- | --- | --- | --- | --- | --- | --- | --- | --- | --- | --- |
| ***K. pneumoniae*** | | | | | | | | | | | |
| 1 | Munoz et al. (2007) | 2006.04 | 10/24 | 41.67% | Clinical | the United States | 36.39 | — | RAPD | — | — |
| 2 | Mekibib et al. (2010) | 2008.11–2009.04 | 5/161 | 3.11% | Subclinical | Ethiopia | 117.9 | Biochem | — | — | — |
| 3 | Zanella et al. (2010) | 2006.01–2007.06 | 22/260 | 8.46% | — | Brazil | 25.6 | Biochem | — | — | — |
| 4 | Ahmed and Shimamoto (2011) | 2008 | 19/99 | 19.19% | — | Egypt | 104.7 | Biochem and Auto | — | Drug resistance | 7/19 |
| 5 | Haftu et al. (2012) | 2009.10–2010.05 | 11/174 | 6.32% | Both^6^ | Ethiopia | 117.9 | Biochem | — | — | — |
| 6 | Timofte et al. (2014) | 2010 | 3/17 | 17.65% | Clinical | the United Kingdom | 281.9 | Auto | — | Drug resistance | 3/3 |
| 7 | Langoni et al. (2015) | 2013 | 6/4,268 | 0.14% | — | Brazil | 25.6 | 16S rDNA | — | — | — |
| 8 | Sudarwanto et al. (2015) | 2011.11–2011.12 | 7/80 | 8.75% | — | Indonesia | 152.6 | — | PFGE | Drug resistance | 8/8 |
| 9 | Diab et al. (2017) | 2015.09–2015.11 | 35/154 | 22.73% | — | Lebanon | 661.7 | MALDI-TOF | PFGE | Drug resistance | 36/36 |
| 10 | He et al. (2017) | 2015 | 2/65 | 3.08% | Clinical | China | 153.8 | MALDI-TOF | PFGE | Drug resistance | 2/2 |
| 11 | Schabauer et al. (2018) | 2015.10–2016.09 | 11/3,020 | 0.36% | — | Austria | 109.7 | 16S rDNA | — | — | — |
| 12 | Wang (2018) | 2017.04 | 13/57 | 22.81% | Clinical | China | 153.8 | Biochem | — | — | — |
| 13 | Hozyen et al. (2019) | 2017.09–2017.12 | 7/24 | 29.17% | Clinical | Egypt | 104.7 | Biochem | — | — | — |
| 14 | Zhou et al. (2019) | 2018.06 | 13/50 | 26.00% | Subclinical | China | 153.8 | Biochem and 16S rDNA | — | — | — |
| 15 | Fuenzalida and Ruegg (2020) | 2016.06–2016.12 | 54/168 | 32.14% | Clinical | the United States | 36.39 | — | PFGE | — | — |
| 16 | Griffioen et al. (2020) | 2017.05–2018.07 | 1/163 | 0.61% | Clinical | the Netherlands | 509.3 | MALDI-TOF | — | — | — |
| 17 | Jiang et al. (2020) | 2019.06–2019.10 | 2/31 | 6.45% | Clinical | China | 153.8 | 16S rDNA | — | — | 2/2 |
| 18 | Cheng et al. (2021) | 2019 | 206/916 | 22.49% | Both | China | 153.8 | Biochem and 16S rDNA | — | Virulence | — |
| 19 | Chung et al. (2021) | 2020.04–2020.09 | 1/234 | 0.43% | Both | Australia | 3.357 | MALDI-TOF | — | — | — |

(*Continued*)

**Supplementary Table 4.** Continued

|  | Reference ID | Sampling time | No. positive/  No. tested | Prevalence | Mastitis  type | Country | Population  density | Detection methods | Genotyping methods | Gene detection | MDR rate |
| --- | --- | --- | --- | --- | --- | --- | --- | --- | --- | --- | --- |
| 20 | Deng et al. (2021) | 2019.09–2019.12 | 51/140 | 36.43% | Clinical | China | 153.8 | 16S rDNA and Auto | — | Virulence | 51/130 |
| 21 | Enferad and Mahdavi (2021) | 2018.04–2018.10 | 80/200 | 40.00% | — | Iran | 52.21 | Biochem | — | Drug resistance | 56/80 |
| 22 | Nobrega et al. (2021) | 2009.12-2011.07 | 28/1,547 | 1.81% | — | Brazil | 25.6 | Biochem | — | Drug resistance | 13/81 |
| 23 | Taniguchi et al. (2021) | 2016.08–2017.07 | 191/1,549 | 12.33% | — | Japan | 345.8 | MALDI-TOF | RAPD | Drug resistance | 22/191 |
| 24 | Tsuka et al. (2021) | 2012.10–2014.12 | 97/1,466 | 6.62% | Clinical | Japan | 345.8 | Auto | PFGE | Drug resistance | — |
| 25 | Yang et al. (2021) | 2017–2018 | 59/857 | 6.88% | Clinical | China | 153.8 | 16S rDNA | — | — | 4/66 |
| ***K. oxytoca*** | | | | | | | | | | | |
| 1 | Zanella et al. (2010) | 2006.01–2007.06 | 3/260 | 1.15% | — | Brazil | 25.6 | Biochem | — | — | — |
| 2 | Ahmed and Shimamoto (2011) | 2008 | 12/99 | 12.12% | — | Egypt | 104.7 | Biochem and Auto | — | Drug resistance | 7/12 |
| 3 | Kateete et al. (2013) | 2010.02–2011.03 | 5/97 | 5.15% | Clinical | Uganda | 235.8 | Biochem | — | — | 2/5 |
| 4 | Xie et al. (2015) | 2013 | 35/593 | 5.90% | — | China | 153.8 | Biochem | — | — | — |
| 5 | Abboud et al. (2021) | 2019.03–2019.04 | 1/90 | 1.11% | — | Lebanon | 661.7 | MALDI-TOF | — | — | 0/1 |
| 6 | Taniguchi et al. (2021) | 2016.08–2017.07 | 6/1,549 | 0.39% | — | Japan | 345.8 | MALDI-TOF | RAPD | Drug resistance | 0/6 |
| ***K. ozaenae*** | | | | | | | | | | | |
| 1 | Zanella et al. (2010) | 2006.01–2007.06 | 2/260 | 7.69% | — | Brazil | 25.6 | Biochem | — | — | — |
| **Unknown Species** | | | | | | | | | | | |
| 1 | Liu (2008) | 2008 | 1/120 | 0.83% | Clinical | China | 153.8 | Biochem | — | — | 0/1 |
| 2 | Botrel et al. (2010) | 2007.01–2008.03 | 17/1,770 | 0.96% | Both^6^ | France | 119.5 | Biochem | — | — | — |
| 3 | Guo (2011) | 2010 | 18/141 | 12.77% | Both | China | 153.8 | Biochem | — | — | — |
| 4 | Kalmus et al. (2011) | 2007–2009 | 87/4,679 | 1.86% | Both | Estonia | 31.26 | Biochem | — | — | — |

(*Continued*)

**Supplementary Table 4.** Continued

|  | Reference ID | Sampling time | No. positive/  No. tested | Prevalence | Mastitis  type | Country | Population  density | Detection methods | Genotyping methods | Gene detection | MDR rate |
| --- | --- | --- | --- | --- | --- | --- | --- | --- | --- | --- | --- |
| 5 | Kamphuis et al. (2011) | 2011 | 4/140 | 2.86% | Clinical | the Netherlands | 509.3 | Biochem | — | — | — |
| 6 | Verbist et al. (2011) | 2008.05–2009.05 | 2/2,644 | 0.08% | Clinical | Belgium | 384.2 | Biochem | — | — | — |
| 7 | Abera et al. (2012) | 2008.10–2009.05 | 1/48 | 2.08% | — | Ethiopia | 117.9 | — | — | — | — |
| 8 | Ba and Li (2012) | 2011.07–2011.08 | 9/64 | 14.06% | — | China | 153.8 | Biochem | — | — | 0/9 |
| 9 | Liu (2012) | 2009.01–2009.12 | 3/50 | 6.00% | Subclinical | China | 153.8 | Biochem | — | — | — |
| 10 | Wang et al. (2012) | 2011.03–2011.04 | 3/76 | 3.95% | Subclinical | China | 153.8 | Biochem | — | — | — |
| 11 | Nam et al. (2013) | 2012.01–2012.11 | 107/2,041 | 5.24% | — | Korea | 527.7 | Biochem | — | — | — |
| 12 | Thompson et al. (2013) | 2007.06–2008.08 | 4/198 | 2.02% | Clinical | Canada | 4.168 | — | — | — | — |
| 13 | Ramírez et al. (2014) | 2009.01–2010.12 | 104/7,954 | 1.31% | Subclinical | Colombia | 46.21 | Biochem | — | — | — |
| 14 | Supre et al. (2014) | 2012.09–2013.09 | 110/27,463 | 0.40% | — | Belgium | 384.2 | Biochem | — | — | 0/59 |
| 15 | Fuenzalida et al. (2015) | 2011.05–2013.11 | 11/279 | 3.94% | Clinical | the United States | 36.39 | Biochem | — | — | — |
| 16 | Koovapra et al. (2016) | 2016 | 154/340 | 45.29% | Both | India | 468.7 | — | — | Drug resistance | 23/291 |
| 17 | Levison et al. (2016) | 2011.04–2012.05 | 18/936 | 1.92% | Clinical | Canada | 4.168 | Biochem | — | — | — |
| 18 | Rodríguez (2016) | 2015.09–2015.12 | 46/142 | 32.39% | — | Peru | 26.06 | — | — | — | — |
| 19 | Gao et al. (2017) | 2014.03–2016.09 | 426/3,288 | 12.96% | Clinical | China | 153.8 | Biochem, 16S rDNA, and Auto | — | — | — |
| 20 | Suleiman et al. (2018) | 2014.01–2014.07 | 79/1,648 | 4.79% | Subclinical | Tanzania | 69.43 | Biochem | — | — | — |
| 21 | Tomazi et al. (2018) | 2014.03–2016.01 | 110/4,212 | 2.61% | Clinical | Brazil | 25.6 | Biochem | — | — | — |
| 22 | Bach et al. (2019) | 2017.02–2017.05 | 6/396 | 1.52% | — | the United States | 36.39 | Biochem | — | — | — |
| 23 | Feng et al. (2019) | 2017 | 32/200 | 16.00% | Clinical | China | 153.8 | 16S rDNA | — | — | 17/32 |
| 24 | Salauddin et al. (2019) | 2019 | 30/48 | 62.50% | Clinical | Bangladesh | 1278 | Biochem | — | — | 30/30 |

(*Continued*)

**Supplementary Table 4.** Continued

|  | Reference ID | Sampling time | No. positive/  No. tested | Prevalence | Mastitis  type | Country | Population  density | Detection methods | Genotyping methods | Gene detection | MDR rate |
| --- | --- | --- | --- | --- | --- | --- | --- | --- | --- | --- | --- |
| 25 | Duse et al. (2021) | 2013.08–2018.12 | 22/755 | 2.91% | Clinical | Sweden | 24.76 | MALDI-TOF | — | — | 0/22 |
| 26 | Fréchette et al. (2021) | 2018–2019 | 56/1,144 | 4.90% | Clinical | Canada | 4.168 | MALDI-TOF | — | — | — |
| 27 | Dyson et al. (2022) | 2011.01–2012.03 | 20/2,572 | 0.78% | — | Australia | 3.357 | Biochem | — | — | — |

*^1^No. postive/No. tested: the ratio of Klebsiella-positive milk samples to total tested milk samples.*

*^2^Population density: unit****—****per sq km.*

*^3^Detection methods: Biochem* ***=*** *biochemical tests; 16S rDNA* ***=*** *16S rDNA identification; MALDI-TOF* ***=*** *MALDI-TOF MS; Auto* ***=*** *automatic analysis systems.*

*^4^Gene detection: molecular characterization of drug resistance genes or virulence genes.*

*^5^MDR rate: the MDR rate of Klebsiella isolates.*

*^6^Both: studies with both clinical mastitis milk samples and subclinical milk sample.*
